# Bamboo-Inspired Morphological Design of Service-Robot Trunks for Xiang–Gan Heritage Museums

**DOI:** 10.3390/biomimetics11100730

**Published:** 2026-10-13

**Authors:** Hua Zhang, Fan Wu

**Affiliations:** School of Packaging Design & Art, Hunan University of Technology, Zhuzhou 412007, China; zhanghua@hut.edu.cn

**Keywords:** biomimetics, bamboo-inspired design, service robot, robot trunk, morphological design, kinematic analysis, structural comparison, Xiang–Gan revolutionary heritage

## Abstract

Revolutionary heritage museums in the Xiang–Gan region provide a bounded context for studying how service-robot trunk morphology can respond to representative needs for height adaptation, interface orientation, and constrained indoor operation. In this setting, a robot’s form is considered alongside its service function as a potential reference to Xiang–Gan red-culture heritage, although visitor interpretation is not evaluated here. This computational design study translates bamboo node–internode segmentation, hollow taper, nodal reinforcement, and compliant bending into explicit morphological, structural, and control variables. Three conditions were defined: a conventional engineering baseline, a culturally styled condition reserved for future evaluation, and a bamboo-inspired coupled condition. Kinematic analysis, linear structural finite-element analysis, and sensitivity analysis were used to compare the baseline and coupled conditions under documented assumptions. The coupled condition produced 74.1% of the baseline composite kinematic coverage index and reduced calculated mass by approximately 7.8%, but it also reduced the calculated bending stiffness by approximately 10.5% and the mass-specific stiffness by approximately 3.0% under the stated linear model. The exploratory analyses indicated 95.7% retention of the baseline feasible composite coverage and a higher index normalized by active-control-variable count, but these quantities are definition-dependent computational sampling results. The findings identify trade-offs in translating bamboo-inspired morphology into a service-robot trunk and highlight joint stiffness, passive extension, and contact behavior as parameters requiring physical calibration. They do not establish physical robot performance, collision safety, cultural effects, or museum deployment.

## 1. Introduction

Public cultural service venues in the Xiang–Gan region—including red-culture museums, revolutionary memorial halls, and former revolutionary sites in Jiangxi and Hunan provinces—present distinct requirements for service robots. Venues such as the Jinggangshan Revolution Museum, the Qiushou Uprising Wenjiashi Memorial Hall, and the Chaling County Workers–Peasants–Soldiers Government Site Memorial Hall serve diverse visitor populations, spanning wide age ranges, body heights, and physical abilities. These venues combine constrained indoor circulation paths, multi-level exhibition layouts, and a cultural mandate to convey revolutionary heritage through the visitor’s entire spatial experience. In such settings, a service robot’s physical form—its trunk morphology, height adaptability, interface orientation, and spatial footprint—directly shapes the quality and accessibility of the human–robot interaction. Yet the morphological design of service robots for these specific regional contexts has received limited systematic attention. Museum guide robots and socially interactive robots provide the relevant interaction context, but their deployment history does not constitute venue-specific evidence for the present design [1,2,3]. The robot is, therefore, considered not only as a mechanical structure but also as a potential element in the interpretation of regional revolutionary heritage; any contribution to visitor understanding remains untested.

Published descriptions of service robots and heritage-museum settings motivate a design question rather than establish measured requirements for every Xiang–Gan venue [4,5,6]. Fixed-height bodies, constrained circulation, and varied visitor stature are, therefore, treated here as contextual considerations to be verified through future fieldwork, not as observed operational facts for the named venues.

Bamboo offers a biological prototype that addresses structurally analogous challenges. The bamboo culm—particularly the node–internode hierarchical organization of *Phyllostachys edulis* and related species—achieves a balance of stiffness, compliance, and mass efficiency through several interconnected structural features [7,8,9,10]. Transverse nodal diaphragms interrupt longitudinal fiber continuity at regular intervals, providing local reinforcement and crack-arresting barriers, while segmenting the culm into modular internode units. The hollow tapered cross-section reduces mass with a controlled penalty in bending stiffness. A radial gradient in vascular bundle density, from high-density outer fibers to low-density inner parenchyma, creates a functionally graded material that distributes mechanical compliance across the wall thickness. Under wind loading, the culm undergoes large elastic deflections without fracture, absorbing energy through passive bending rather than active resistance. In the Xiang–Gan regional context, bamboo—particularly Jinggang bamboo (Jinggangshan zhuzi) cultural imagery—carries additional symbolic resonance as a material associated with revolutionary perseverance and regional identity, though this cultural dimension is treated as a contextual framing rather than a biomechanical claim in the present study.

These biological features have motivated engineering translation, including bamboo-inspired segmented tubes and exoskeleton structures [11,12]. Related work on craft-mediated biomimetic translation and cross-disciplinary evaluation treats the move from a biological or cultural analogue to engineering criteria as an explicit and reviewable process [13,14]. Studies of traditional-craft development, cultural-pattern design, sensory evaluation, perceptual experience, and AI-assisted biomimetic design address distinct design and user-facing outcomes [15,16,17,18,19]. These literatures provide context but do not validate the engineering performance evaluated here. At the systems level, soft-robotics research establishes the relevance of compliant, segmented bodies, and controllable stiffness [20,21,22,23,24,25,26,27,28], while morphological-computation research motivates joint consideration of body and controller without equating passive mechanics to demonstrated intelligence [29,30,31,32]. For museum and heritage settings, telepresence, educational interaction, cultural-experience design, and context-aware guide-robot studies provide application context rather than mechanical validation [33,34,35,36].

Within the verified literature considered in this study, three gaps motivate the present work. First, bamboo biomimetics has produced documented material, structural-component, and exoskeleton examples, but limited work explicitly links bamboo mechanisms to a reviewable set of service-robot-trunk variables and evaluates those variables under a controlled computational comparison. Second, morphological-computation, compliant-robotics, and continuum-robotics research provides concepts for coupling body and control but does not by itself specify how a particular biological mechanism should be translated into engineering variables, comparison conditions, and measurable metrics for a museum service robot. Third, museum- and heritage-robot studies primarily address interaction, navigation, content delivery, and engagement, leaving trunk morphology, height and orientation adaptation, structural mass and stiffness, and passive coupling as application-specific design questions. These gaps are framed as limited explicit links within the verified literature, not as claims that no related study exists.

This study specifies a bamboo-inspired morphology–structure–control (MSC) translation framework for a segmented service-robot trunk. It maps four biological mechanisms to engineering variables, measurable computational metrics, and comparison conditions. All conditions use four physical segments and three inter-segment interfaces. Condition A is the conventional rigid computational baseline; Condition B is technically identical to A in the present model and reserved for future cultural-semantic evaluation; and Condition C has two independently commanded active control variables and one passively coupled interface in the kinematic abstraction.

The contributions of this manuscript are: (i) a traceable biological-mechanism-to-engineering-variable translation framework connecting bamboo mechanisms with explicit robot-trunk variables; (ii) a controlled A/B/C comparison architecture, separating the engineering baseline, a future cultural-semantic treatment, and the bamboo-inspired coupled morphology; (iii) a computational evaluation of the resulting trade-offs, using kinematic coverage, sampled interference-feasibility frequency, linear structural finite-element outputs, and sensitivity analyses; and (iv) an evidence-bounded identification of parameters requiring physical calibration, rather than a claim of demonstrated superiority. The results are not evidence of a validated robot, service outcome, cultural effect, or final design selection.

## 2. Background and Related Work

### 2.1. Bamboo Biological Structures and Biomimetic Translation

Bamboo belongs to the subfamily Bambusoideae (*Poaceae*) and comprises over 1600 species distributed across tropical, subtropical, and temperate zones. Despite this diversity, several structural features are conserved across bamboo species and have been the subject of systematic biomechanical characterization. The bamboo culm is organized as a hierarchical composite: at the macroscopic scale, transverse nodes segment the culm at regular intervals, with internode lengths and wall thicknesses varying along the culm height in a species-specific pattern. At the mesoscale, the culm wall consists of vascular bundles—cellulose fiber-reinforced structures—embedded in a parenchyma matrix, with the volume fraction of vascular bundles decreasing from the outer wall (high density) to the inner wall (low density), forming a functionally graded material (FGM). At the microscale, cellulose microfibrils within the fiber walls are oriented at characteristic angles that govern local stiffness and fracture behavior. Comparative studies of node, internode, culm-height, and vascular-bundle variation support treating these relationships as species- and specimen-dependent rather than as universal dimensional prescriptions [37,38,39,40].

Han et al. (2024) [7] provided a systematic review of bamboo mechanical behavior and its biomimetic applications, documenting four key structure–function relationships. First, the node–internode hierarchical organization: nodes interrupt longitudinal fiber continuity with transverse vascular bundles, creating barriers to crack propagation, while locally increasing wall thickness; internodes maintain continuous axial fiber alignment for efficient load bearing. Second, the hollow tapered culm: the circular hollow cross-section maximizes the second moment of area per unit mass, achieving bending stiffness with minimal material; the taper from base to tip reduces self-weight at the cantilevered upper portion. Third, the radial FGM: the graded distribution of fiber density through the wall thickness creates a stiffness profile that resists ovalization under bending and distributes contact stresses. Fourth, the large-deflection elastic recovery: under wind or snow loading, the culm undergoes elastic bending well beyond the linear regime without structural failure, recovering its original shape upon unloading. These four features operate as an integrated system—the hollow taper reduces mass, the FGM wall resists local buckling, the nodes arrest longitudinal cracks, and the global compliance absorbs transient loads—rather than as independent design features. The present abstraction uses these relationships as design principles, not as direct material-property inputs [7,8,9,10].

Several authors have cautioned against treating bamboo biomechanical data as universally transferable across species, growth conditions, and measurement protocols. Node spacing, wall thickness, taper rate, and FGM profiles vary substantially between *Phyllostachys edulis* (Moso bamboo), *Bambusa vulgaris*, *Guadua angustifolia*, and other commercially and structurally significant species. Measured mechanical properties—the elastic modulus, bending strength, and fracture toughness—depend on the moisture content, age at harvest, position along the culm, and testing configuration. These sources of variation imply that biomimetic translation from bamboo should abstract the structural principles (node–internode segmentation, hollow taper, FGM wall, and global compliance) rather than directly replicate species-specific dimensional ratios. In this study, *Phyllostachys edulis* is used only as a literature-based biological reference for generic bamboo biomechanics; it is not identified as the local species represented by the term “Jinggang bamboo.” The latter is treated strictly as Xiang–Gan red-culture imagery. The four structural principles are, therefore, translated into engineering design variables independently of any assumed local species identity, consistent with reported variation in node/internode and vascular-bundle behavior across bamboo specimens [37,38,39,40].

### 2.2. Bamboo-Inspired Robotics and Adaptive Structures

The translation of bamboo structural features into robotic and structural systems has produced several documented precedents. Zahedi et al. (2023) [11] developed BiEXO, an upper-limb exoskeleton in which carbon-fiber-reinforced polymer tubes (analogous to bamboo internodes) are connected by aluminum nodes (analogous to bamboo nodes), with cables routed internally through the hollow tubes. Finite element analysis and human-subject wear trials demonstrated that the hollow tubular architecture achieved a favorable strength-to-weight ratio, with the arm assembly weighing approximately 800 g. The BiEXO design explicitly references the bamboo node–internode pattern as its biomimetic source and validates the structural efficiency of hollow segmented tubes with localized nodal reinforcement. However, BiEXO was designed as a wearable exoskeleton for upper-limb rehabilitation, not as a service-robot trunk; its design priorities (lightweight wearability and trajectory tracking for shoulder and elbow joints) differ from those of a museum service robot (height-adaptive coverage, multi-directional interface orientation, and collision-free workspace under spatial constraints). The documented BiEXO precedent is particularly relevant as a component-level translation, while its wearable task remains distinct from a museum trunk [11].

Additional bamboo-node actuator reports were not used as evidentiary sources because their bibliographic records were not independently verified. The present argument, therefore, relies on the verified bamboo-inspired structural and soft-robotics literature cited above.

Beyond explicitly bamboo-titled work, compliant, variable-stiffness, and continuum-robot research provides engineering context for segmented bodies [25,26,27,28]. These studies establish transferable design concerns, not proof of bamboo-specific performance or museum-task suitability.

The verified BiEXO study demonstrates a component-level translation of bamboo-inspired hollow tubular links [11]. The present study does not claim to be the first bamboo-inspired robotic structure or to surpass prior work; it instead tests a service-robot trunk comparison question under stated computational conditions.

### 2.3. Embodied Intelligence and Morphological Computation

The concept that a robot’s physical morphology can contribute to behavior has been formalized through morphological computation and morphology–control co-design [29,30,31,32]. These accounts stress feedback, co-design, and task-specific embodiment; they do not equate a passive mechanism alone with validated embodied intelligence.

Recent morphology–control research supports treating body parameters and control policy as coupled design variables, but it does not replace a controlled comparison under a defined protocol [31,32].

In the present study, the term "morphology–structure–control (MSC) coupling" is used with a specific operational meaning: the modeled trunk structure (taper, node rings, and wall-thickness gradient) is examined for its contribution to composite kinematic coverage and structural load bearing, with one passively coupled interface in Condition C. Compliant segments are considered only as an untested future design concept. The separately reported kinematic metric counts independently active inter-segment interfaces (*n*_ind_ = 3 for A/B and *n*_ind_ = 2 for C). The MSC framework proposed here is an application-specific operationalization of the general morphological computation principle, not a claim to have invented the concept. The present study does not use the term "embodied intelligence" to describe its contribution, because that term conventionally implies a physically realized robot exhibiting adaptive behavior through perception–action loops—evidence that the current stage of this work (computational kinematic and linear structural analysis) does not provide.

### 2.4. Adaptive Service Robots in Museum and Heritage Environments

Service robots in museums and cultural-heritage sites have been studied through telepresence and remote accessibility, social and educational engagement, cultural-experience design, and context-aware guidance [33,34,35,36]. These studies establish application context, but they do not validate the present trunk geometry or its structural performance.

Public reports describe digital interpretation and robotic guidance initiatives in Chinese red-culture museum settings. They are treated here as contextual background only and do not establish physical-robot deployment, demand, or mechanical validation at the three focal Xiang–Gan venues.

The design requirements considered in the present study—visitor height variation, multi-directional interface orientation, spatial constraints of exhibition halls, and regional cultural resonance—are derived from published descriptions of the three core venues and from general principles of accessible museum service design. They are representative design requirements, not field-validated measurements. No systematic observational study of visitor–robot interaction has been conducted at any of the three venues for the purposes of this research. The museum-oriented design considerations are linked to the computational model as follows: visitor-stature variation is represented by effective trunk-height range (*ΔH*); interface orientation for explanation and interaction is represented by orientation range (*Δθ*); constrained indoor configuration is represented by segment geometry and coupling configuration and evaluated through a sampled interference-feasibility frequency; lightweight service structure is represented by the taper, wall thickness, and node-ring geometry, and evaluated through mass, bending stiffness, and mass-specific stiffness; and future contact accommodation is represented by compliance and passive-joint behavior, for which contact-force and physical-joint-calibration metrics remain untested. These are representative design considerations and computational proxies, not field-measured requirements or collision-safety evidence.

The Xiang–Gan region—the border area of Jiangxi and Hunan provinces—holds a distinctive position in the history of the Chinese revolution. It was the location of the Jinggangshan revolutionary base area (1927–1930), the site of the Autumn Harvest Uprising (1927), and the birthplace of the first Workers–Peasants–Soldiers Government in Chaling County (1927). These historical events have left a dense network of revolutionary heritage sites, including the Jinggangshan Revolution Museum (a national first-class museum), the Qiushou Uprising Wenjiashi Memorial Hall (a nationally protected major historical site), and the Chaling County Workers–Peasants–Soldiers Government Site Memorial Hall (a county-level protected site). The three venues span a range of institutional scales, exhibition formats (indoor museum, historical site, and memorial hall), and visitor demographics, from organized school groups to independent adult visitors.

Regional policy and cultural-development discussions provide contextual motivation for intelligent interpretation services. They are not used here as measurements of visitor demand, robot performance, or venue-specific operating conditions.

Bamboo carries specific cultural resonance in this regional context. Jinggang bamboo (Jinggangshan zhuzi) appears in revolutionary cultural narratives as a symbol of resilience, adaptability, and rootedness—qualities associated with the revolutionary base area’s survival under blockade. This cultural imagery is distinct from the biomechanical properties of any particular bamboo species and is treated in this study as a contextual design motivation, not as a source of engineering parameters. The design principle of “structure as cultural expression”—in which the bamboo-inspired structural logic of the trunk (taper, node rings, and segmented proportion) simultaneously serves engineering function and conveys regional cultural identity—is proposed as a framework for integrating cultural context into biomimetic morphological design. This principle is treated as a design intent; its effectiveness in conveying cultural meaning to visitors has not been empirically evaluated and is reserved for future user studies.

The preceding sections establish a bounded rationale: bamboo biomechanics supplies abstract structural principles; verified robotics research supplies the component- and systems-level context; and museum-robot studies supply the application context. None validates the present trunk geometry or substitutes for venue-specific testing.

This study addresses this intersection by translating four bamboo mechanisms into stated engineering variables, defining three trunk conditions, and comparing A and C under documented computational regimes. It reports the complete frozen kinematic and linear-structural result set, including negative H1 and H2 model outcomes. The Xiang–Gan Museum context motivates the design frame, but is not treated as a field-validated operational dataset. No physical prototype, contact-safety test, or superiority claim is made.

## 3. Materials and Methods

**Figure 1:**
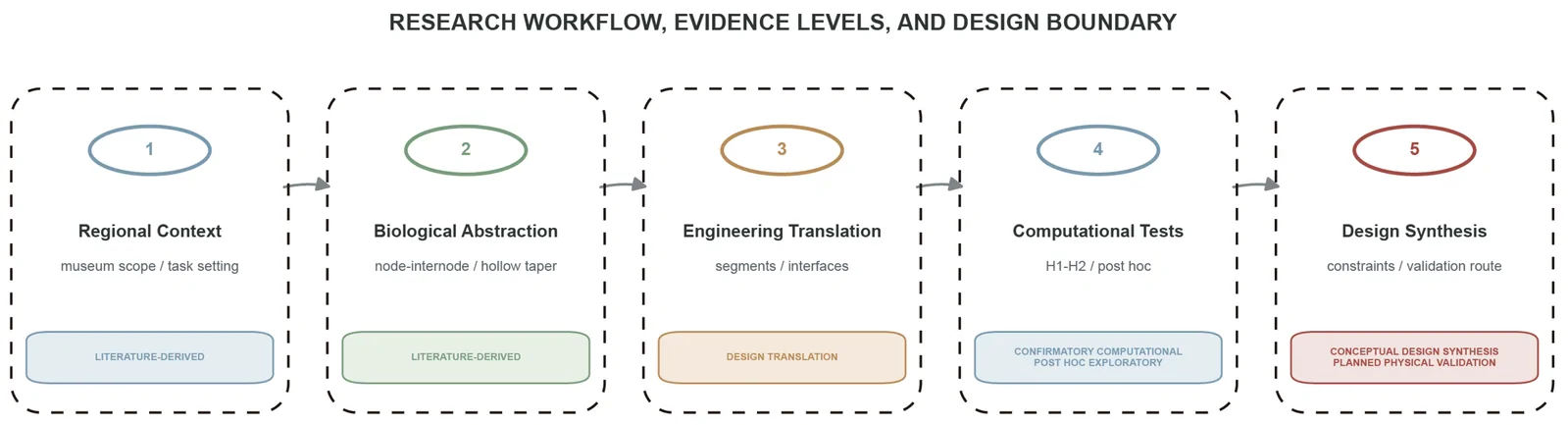
The research workflow, evidence levels, and design boundary. The regional context and biological mechanisms are literature-derived; controlled computations distinguish the protocol-defined and post hoc evidence; and the final stage is an evidence-constrained conceptual design synthesis with planned physical validation.

The overall computational workflow and evidence boundaries are summarized in Figure 1.

### 3.1. Study Scope and Evidence Classification

This study addresses the morphological design of a segmented service-robot trunk for public cultural service applications in Xiang–Gan red-culture museums. The geographic scope is bounded to Jiangxi and Hunan provinces’ red-culture heritage venues, with three anchor sites providing representative service scenarios: the Jinggangshan Revolution Museum (Jiangxi, national first-class museum), the Qiushou Uprising Wenjiashi Memorial Hall (Hunan, nationally protected historical site), and the Chaling County Workers–Peasants–Soldiers Government Site Memorial Hall (Hunan, county-level protected site). Service task scenarios—including guided tours, exhibit explanation, visitor height adaptation, and multi-user interface orientation—are derived from published descriptions of these venues and from general principles of accessible museum service design. These scenarios are representative design requirements; they have not been validated through systematic on-site observation or structured interviews with museum staff. The bounded workflow and evidence levels are summarized in Figure 1.

The present study is a computational design investigation. Its scope is limited to: (i) the abstraction of biological mechanisms from the published bamboo biomechanics literature; (ii) the translation of these mechanisms into engineering design variables for a segmented robot trunk; (iii) protocol-defined composite kinematic coverage analysis under two comparison regimes; (iv) linear static structural finite element analysis under two structural comparison regimes; and (v) parameter sensitivity analysis through single-factor sweeps and Latin-hypercube sampling. This study does not include physical prototype fabrication or testing, nonlinear contact analysis, user studies, cultural perception experiments, or field deployment in any museum venue. Joint rotational stiffness calibration (designated J2) has been designed as a planned protocol but has not been executed. All structural loads are provisional engineering estimates, not values measured from operational museum environments.

The research workflow has five stages: a literature and evidence review; biological mechanism abstraction; engineering translation and MSC specification; computational kinematic and linear-structural analysis; and planned physical validation. The computational stages use the documented model settings, 63 completed Run 2 solver jobs, a 200-sample expanded post hoc LHS analysis, and 56 mesh-convergence checks. J2 is reserved for future physical calibration and has not been executed in the present study.

The evidence is classified as literature-derived, analytical, computational, exploratory/post hoc, or planned physical validation. These labels describe the origin and scope of evidence; they do not imply external registration, independent replication, or field validation.

### 3.2. Bamboo Mechanism Abstraction

**Figure 2:**
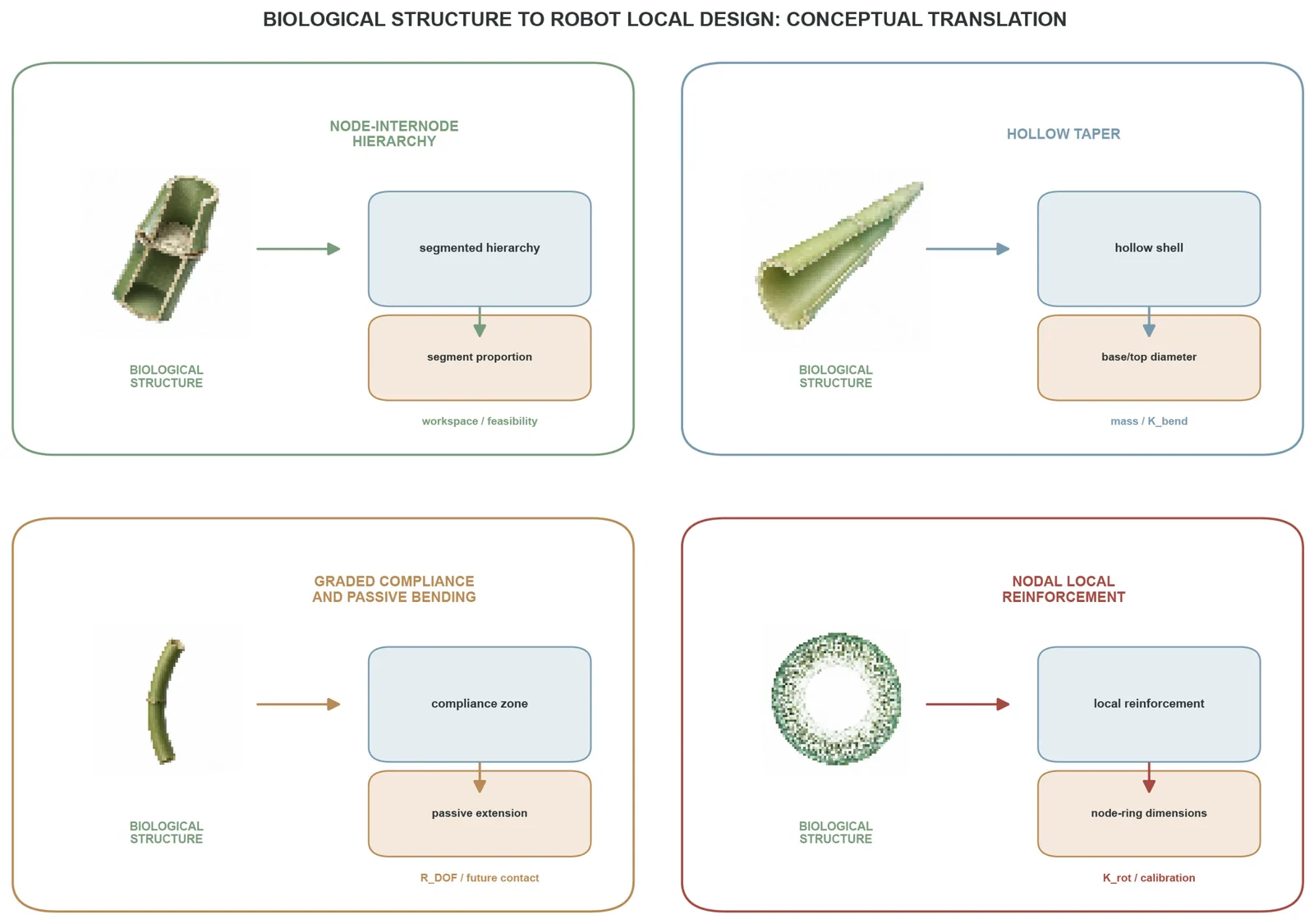
The biological-to-engineering mechanism translation. Each module links a conceptual biological structure to a robot-local abstraction and one key design variable or metric. The diagram is a conceptual translation only; the biological illustrations are not measured input data. Colors are used only to visually distinguish the conceptual mechanism modules and do not encode quantitative values, evidence levels, or performance.

Four structural mechanisms were abstracted from the published bamboo biomechanics literature and classified by their functional role in the target robot trunk system. Their translation is summarized in Figure 2.

Core Mechanism A—Node–Internode Hierarchical Segmentation (M01). Bamboo nodes segment the culm and provide local reinforcement, while internodes maintain structural continuity. The engineering abstraction is a four-segment robot trunk with three inter-segment interfaces. In the computational comparison, A/B have three independently active interface variables, and C has two, with one C interface passively coupled. This mapping motivates composite kinematic coverage and structural metrics but does not imply independent actuators for every segment or validated hardware.

Core Mechanism B—Graded Compliance and Passive Bending (M03 + M05). Biological structure: the bamboo culm wall exhibits a radial gradient in vascular bundle density, from high-density outer fibers (high stiffness) to low-density inner parenchyma (low stiffness), forming a functionally graded material. Under wind loading, the culm undergoes large elastic deflections without fracture, absorbing energy through passive bending. Abstracted principle: a stiffness gradient through the structural cross-section, combined with global elastic compliance, enables passive energy absorption and reduces peak forces during transient loading. Engineering translation: a future hardware translation could incorporate a compliant segment, for example using a lower-modulus material, such as thermoplastic polyurethane (TPU), alongside rigid segments. Any implementation would remain a future design hypothesis rather than a result of the present computational model. No TPU segment, nonlinear contact response, or contact-force attenuation is modeled or validated in the present study. Prospective robot function: a future concept for physical investigation, not a demonstrated contact-safety function. No contact-force or safety outcome is evaluated here.

Auxiliary Mechanism—Hollow Tapered Lightweighting (M02). Biological structure: the bamboo culm is a hollow circular cylinder with a taper from base to tip; the hollow cross-section maximizes the second moment of area per unit mass, and the taper reduces self-weight at the cantilevered upper portion. Abstracted principle: a hollow, thin-walled circular cross-section with controlled external taper achieves bending stiffness with reduced structural mass. Engineering translation: the trunk segments employ a hollow circular cross-section with a taper ratio of 0.15 (diameter reduction from 100 mm at the base to 85 mm at the top); the wall thickness is specified independently of the diameter to allow mass–stiffness trade-off analysis. Expected robot function: a reduced total structural mass while maintaining adequate bending stiffness for service payloads. Measurable performance: the total mass (*M_t_*_o*t*al_), bending stiffness (*K*_bend_), and specific stiffness (*K*_bend_/*M_t_*_o*t*al_).

Auxiliary Design Principle—Nodal Local Reinforcement (M04). Biological structure: at bamboo nodes, the wall thickness increases locally, and transverse vascular bundles form a three-dimensional network, providing resistance to transverse splitting and ovalization. Abstracted principle: local thickening at segment interfaces can reduce stress concentration without proportionally increasing the total mass. Engineering translation: each segment interface includes a node ring—a localized increase in wall thickness (from 4 mm to 6 mm) over a 25 mm axial length. Expected robot function: a reduced stress concentration at segment connections. Measurable performance: peak von Mises stress at joint regions and parameter sensitivity (contribution to global displacement < 2.5% in elastic analysis).

The translation pathway from the biological structure to engineering variables follows a five-level mapping: L1 (biological form) → L2 (structural embodiment) → L3 (sensing and control) → L4 (service behavior) → L5 (cultural effect). The mapping is a conceptual design framework; the service and cultural effects associated with its later levels have not been validated in this study. The functional classification (core vs. auxiliary) reflects whether the mechanism directly produces a measurable service adaptation behavior (core) or serves as an enabler or quality-assurance measure for the core mechanisms (auxiliary).

### 3.3. MSC Framework and Comparison Conditions

The morphology–structure–control (MSC) framework operationalizes the principle that a robot’s physical form (morphology), its structural properties (structure), and its actuation and sensing strategy (control) are coupled design dimensions rather than independent modules. In the present application, the MSC coupling takes the following specific form: the trunk morphology (segmented architecture with taper and node rings) determines the structural mass distribution and stiffness profile, which in turn determines how much of the composite kinematic coverage and structural load-bearing can be achieved through passive geometric configuration rather than through active control. All conditions retain four physical segments and three inter-segment interfaces. The kinematic metric counts independently active interfaces (*n*_ind_ = 3 for A/B and *n*_ind_ = 2 for C); the remaining C interface is passively coupled and contributes to coverage extension and structural compliance through its fixed geometric relationship.

The MSC framework is not claimed to be novel or validated. In this manuscript, it is a specified design hypothesis subjected to initial computational testing. H1 and H2 were not supported under the frozen comparison models; the other proposed effects require independent future tests.

**Figure 3:**
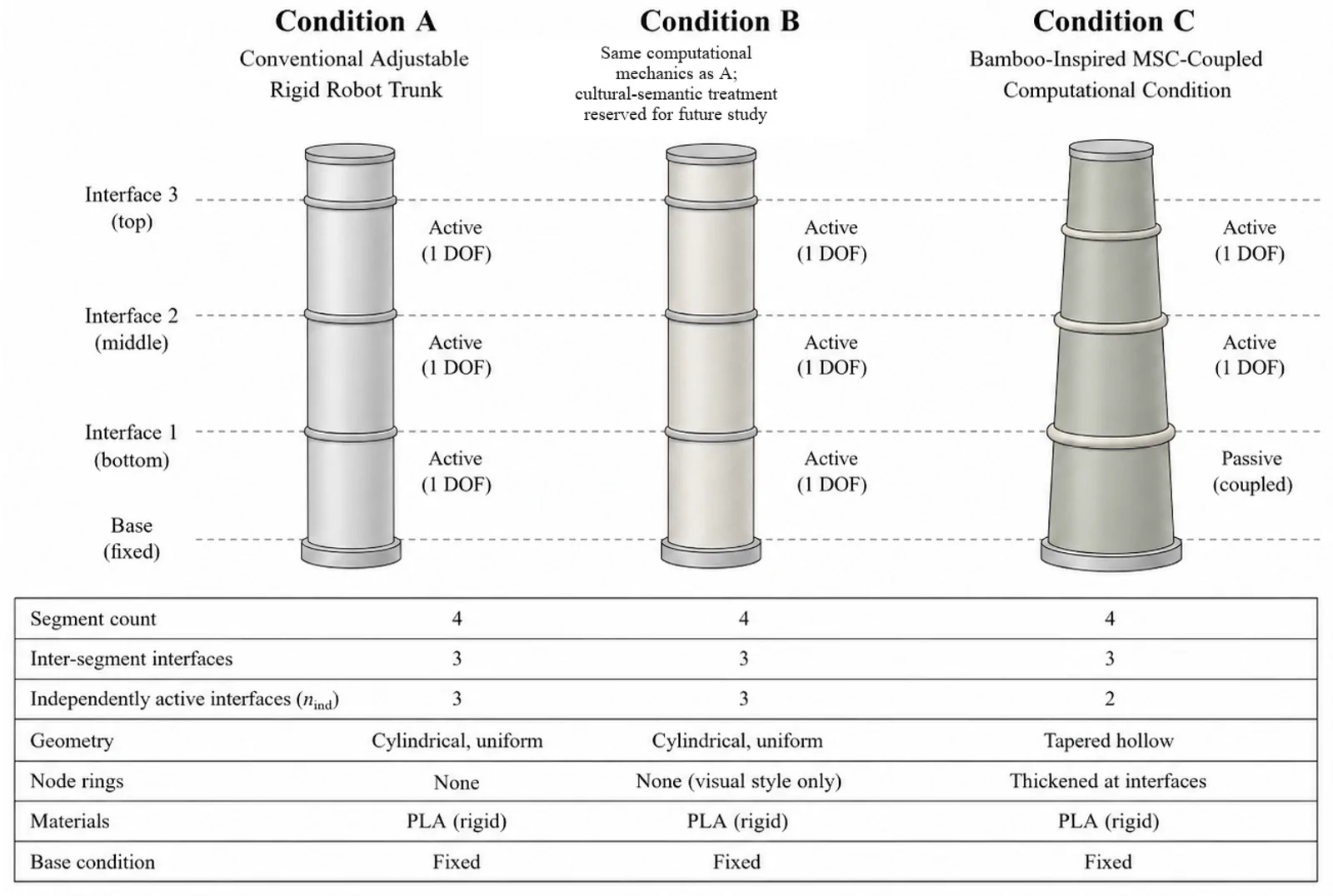
Controlled computational Conditions A, B, and C. All conditions retain four segments and three interfaces. A has three independently active interfaces, B uses the same computational mechanics as A, with future cultural-semantic treatment only, and C has two independently active plus one passive-coupled interface. The diagram is based on frozen computational geometry and is not a physical prototype.

Three trunk conditions are defined to isolate the effect of MSC coupling from cultural surface treatment. All conditions share a common segment count (*n* = 4), total trunk height (500 mm for the computational structural model), base boundary condition (fully fixed at the bottom face), and material class (PLA for rigid segments). The three conditions are compared schematically in Figure 3.

Condition A—Conventional Adjustable Rigid Robot Trunk. Four identical cylindrical segments, each with an outer diameter of 100 mm and a wall thickness of 4 mm, constructed from homogeneous PLA. The condition uses the equal-hardware-budget actuation arrangement; for kinematic comparison, it has three independently active inter-segment interfaces (*n*_ind_ = 3). Segment interfaces are rigid flange connections with zero passive deformation. This condition represents the conventional engineering approach: all adaptive function is supplied by active control; the structure serves only as a load-bearing housing. Condition A serves as the engineering performance baseline.

Condition B—Bamboo-Styled Conventional Robot Trunk. In the present computational model, Condition B is technically identical to Condition A. A future cultural-semantic treatment may use removable surface color, graphics, texture, or other visual treatment; no added mass, stiffness contribution, altered external envelope, or distinct numerical result is asserted in this manuscript. Condition B was not entered as a separate kinematic or structural computation and is reserved solely as a future cultural-semantic comparison condition (H5 not tested).

Condition C—Bamboo-Inspired MSC-Coupled Computational Design Condition. The computational geometry has four segments with a tapered hollow profile (an outer diameter of 100 mm at the base and 85 mm at the top; a taper ratio of 0.15), a 4 mm wall thickness, and node rings locally thickened to 6 mm over 25 mm at the interfaces. Two of the three interfaces are independently active in the kinematic metric (*n*_ind_ = 2); the remaining interface is passively coupled using a provisional coupling factor of 0.3 and passive extension range of 5°. Both values were adopted as nominal engineering-model settings for the initial computational comparison; neither was derived from measured bamboo biomechanics or calibrated physical-joint data. The expanded post hoc sensitivity analysis treats these values as assumptions to be tested across the recorded ranges rather than as validated or biomimetically optimal parameters. A compliant-zone concept is reserved for future physical work and is not represented as a separate material or contact model in the reported linear FEA.

The comparison between A and C is governed by explicit comparison regimes (Section 3.5). Variables that differ between A and C—mass, wall thickness distribution, effective bending stiffness, and active DOF count—are consequences of the MSC design strategy and are reported transparently rather than treated as confounds to be eliminated.

### 3.4. Kinematic Model and Metrics

The kinematic abstraction represents the trunk as four serial physical segments joined by three inter-segment interfaces. The base support is not counted as an inter-segment interface. The metric *n*_ind_ counts independently commanded active interface variables, not physical joints, scalar pitch/yaw/height coordinates, or future actuators. A/B use *n*_ind_ = 3; C uses *n*_ind_ = 2, with the remaining C interface passively coupled. Height and orientation ranges are provisional model assumptions rather than measured hardware limits.

For condition X, a composite kinematic coverage index, *Ω*_X_, was defined according to Equation (1) as the product of effective height range, effective orientation range, and the number of independently active interface variables. The index is named a composite kinematic coverage index to distinguish it from a geometric task-space volume.

(1) $$\Omega_{X}={\Delta H}_{X}\times {\Delta \theta }_{X}\times n_{ind,X},\;\;\;\;X\;\in \;\{A,\;C\}$$

In Equation (1), *ΔH* is expressed in centimeters, *Δθ* in degrees, and *Ω* in cm·°. Because cm·° is a composite unit rather than a conventional physical-workspace unit, *Ω* is not a three-dimensional reachable-volume measurement or a direct measure of the physical robot workspace. It is a protocol-defined comparative quantity used only to compare the stated height range, orientation range, and independently active interface count within the present kinematic abstraction. At the nominal settings, *ΔH*_A_ = *ΔH*_C_ = 4.4 cm, *Δθ*_A_ = 90°, *Δθ*_C_ = 100°, *n*_ind,A_ = 3, and *n*_ind,C_ = 2. Equation (2) defines the feasible composite kinematic coverage from the sampled interference-screening fraction, while Equations (3) and (4) define the exploratory retention and per-active-control-variable normalized indices, respectively.

(2) $$\Omega_{feasible,X}=f_{X}\times \Omega_{X},\;\;\;\;f_{X}=\frac{N_{feasible,X}}{200}$$

(3) $$R_{retention}=\frac{\Omega_{feasible,C}}{\Omega_{feasible,A}}$$

(4) $$R_{DOF}=\frac{\frac{\Omega_{feasible,C}}{n_{ind,C}}}{\frac{\Omega_{feasible,A}}{n_{ind,A}}}$$

Height and orientation were obtained from the frozen forward-kinematic parameterization. The 1° pitch/yaw discretization is a computational sampling resolution, not a physical measurement; the primary *Ω* metric itself follows Equation (1).

Self-interference was screened using the frozen cylindrical minimum-distance routine across all unordered segment pairs, not adjacent pairs only. A sampled configuration is classified as feasible when every tested pair meets the provisional clearance threshold. The feasible fraction *f_X_* and the resulting feasible composite kinematic coverage are model-derived computational screening quantities and do not represent hardware contact testing or physical collision-safety evidence.

*Ω* is the protocol-defined primary composite kinematic coverage index. Feasible composite kinematic coverage, *R*_re*t*en*t*ion_, and the per-active-control-variable normalized index are exploratory model outputs defined above. No independent empirical validation is claimed.

Joint actuation effort was examined according to Equation (5), using the following trajectory-sum metric:

(5) $$J_{\theta }\;=\;\Sigma_{t}\;\Sigma_{j}\;|{\Delta \theta }_{active,j,t}|$$

In Equation (5), *t* indexes trajectory samples, *j* indexes active interface variables, and *Δθ*_ac*t*ive,*j*,*t*_ is the active angular increment for interface *j* at sample *t*. *J_θ_* was computed from synthetic joint trajectories because physical joint-angle measurements were unavailable. It is a synthetic computational check and does not validate a hardware actuation-effort claim.

### 3.5. Comparison Regimes

The A/C comparison is organized into two kinematic and two structural protocol-defined regimes.

Kinematic Regime 1—Equal Actuator Budget. The model compares A and C with the same four conceptual segments and a protocol-defined equal-actuator-budget framing; this is not a claim of identical physical joint count or assembled hardware. In the kinematic abstraction, A has *n*_ind_ = 3, and C has *n*_ind_ = 2, with one passively coupled interface. Taper geometry and passive coupling are design differences reported transparently.

Kinematic Regime 2—Equal Functional Requirement. This is a planned future comparison framework only. No separate numerical result is reported for it in this manuscript, and its task requirements, hardware constraints, and acceptance criteria would require a prospectively specified protocol before execution.

Structural Regime S1—Equal External Envelope and Payload. Conditions A and C share the same external envelope diameter at the base, total height, base boundary conditions, interface dimensions, payload magnitude and position, support conditions, material class, and maximum working posture. This regime isolates the effect of internal structural organization (taper, node rings, and wall thickness gradient) on mass, stiffness, and displacement under identical external geometric constraints and loading.

Structural Regime S2—Equal Structural Mass. The total structural mass of Condition C is normalized to that of Condition A by uniformly scaling the wall thickness (scale factor *s* = 1.0883, derived from the mass ratio *M*_A_/*M*_C_ at nominal wall thickness). All other variables retain their S1 values. This regime tests whether, under equal mass investment, the tapered, node-ring-reinforced geometry of Condition C achieves stiffness comparable to the uniform cylindrical geometry of Condition A.

Variables that cannot be simultaneously equalized between A and C are reported explicitly. Condition C differs through the taper, node rings, wall-thickness distribution, and passive coupling; these coupled differences are part of the tested design condition rather than isolated causal factors. The future compliant TPU concept was not included in the reported linear structural model.

### 3.6. Latin-Hypercube, Sensitivity, and Sample-Size Stability Analysis

The expanded post hoc robustness analysis used a 200-sample Latin-hypercube design with random seed 20260718. The frozen sampled parameters were the coupling factor (0.05–0.50), passive extension (0.5–15.0°), joint-range scale (0.5–1.5), segment count (integer 2–6), and collision clearance (5–50 mm). The original 200-sample post hoc results were retained unchanged. As a separate revision-requested stability check, the nominal A/C geometry was held fixed while the original feasibility sampler was repeated at *N* = 50, 100, 200, 500, and 1000 configurations with 20 seeds per sample size (100 runs total; seeds 2026091301–2026091320).

The Latin-hypercube analysis and the repeated-seed sample-size check were added after the primary comparison and are descriptive robustness or stability explorations, not prospectively specified or protocol-defined tests. Their percentages and summary intervals describe the sampled parameter space or repeated computational samples and should not be interpreted as population probabilities, independent validation, or statistically proven convergence.

The Latin-hypercube outputs are reported as counts and frequencies within the stated 200-sample computational design. The sample-size stability outputs are reported as means, medians, standard deviations, ranges, and empirical 2.5–97.5th percentiles across 20 seeds at each N. No unregistered universal threshold is used to label these frequencies as robust, suggestive, or statistically protocol-defined.

### 3.7. Structural Model and Boundary Conditions

The structural analysis is a frozen linear-elastic computational subsystem model using CalculiX 2.22 and C3D8I elements. It represents a 500 mm trunk section with the stated idealizations and a medium mesh that passed the recorded convergence checks; it is not a validated full robot, material qualification, or physical safety analysis. The model represents rigid PLA shell/subsystem behavior, a small-deformation linear-elastic comparison, and the J0 structural response under the stated loads. It does not represent nonlinear TPU compliance, large-deflection behavior, passive-joint contact or friction, backlash, tendon/cable compliance, human contact, dynamic impact, or assembled-robot collision safety.

Geometry, mesh, and boundary conditions. Condition A: four uniform hollow cylinders (*D*_o_ = 100 mm, *t*_wall_ = 4 mm) connected by rigid tie constraints at segment interfaces. Condition C: four tapered, hollow cylinders (*D*_o_ = 100 mm at base, linearly tapering to 85 mm at top, taper ratio 0.15; *t*_wall_ = 4 mm) with node rings (*t*_wall_ = 6 mm over 25 mm axial length) at each interface. Both conditions were meshed with structured hexahedral grids; three mesh levels were defined—coarse (~3500 elements), medium (7104 elements, the formal comparison level), and fine (~14,000 elements)—for mesh convergence assessment. The base face of the lowest segment was subjected to a fully fixed boundary condition (all translational degrees of freedom fixed for the solid-element model). Segment interfaces in the primary (J0) analysis were modeled as rigid tie connections (infinite joint stiffness). The 500 mm domain is a controlled structural subsystem used for the A/C comparison. It is not a geometrically scaled representation of the approximately 1300 mm conceptual whole-product envelope. No direct extrapolation of displacement, bending moment, buckling response, or stress from the 500 mm subsystem to the full-height concept is made in this study.

### 3.8. Material, Load, and Output Assumptions

Material and load assumptions. Rigid segments were modeled as isotropic PLA with a provisional elastic modulus *E* = 3.0 GPa (sensitivity range 2.5–3.5 GPa), Poisson’s ratio *ν* = 0.33, and density *ρ* = 1240 kg/m^3^. These are engineering model inputs, not measurements of a fabricated specimen. The compliant TPU concept was not modeled. Loads and eccentricities are provisional engineering assumptions rather than values measured in a museum or physical robot.

LC1–LC4 are baseline static service cases; LC5–LC6 are load-boundary static cases; LC7–LC8 are linear buckling cases; and LC9–LC13 are static sensitivity studies. The supplemental SC-BEND calculation provides a bending-stiffness quantity. Sensitivity studies are not additional independent service load cases.

Output metrics. For each converged job, the following were extracted from the CCX.dat output file: the maximum displacement magnitude at the top surface (*δ*_in*t*erface_), peak von Mises stress in a representative region, excluding the fixed-base stress concentration zone, total strain energy, the total reaction force and moment at the fixed base, and the first positive buckling eigenvalue (*λ*_1_). The bending stiffness *K*_bend_ was computed from the load case SC-BEND as the ratio of the applied transverse load to the resulting transverse displacement at the load application point.

### 3.9. Solver Validation and Mesh Convergence

The solver and meshing workflow were checked with three benchmark problems at three mesh densities (nine jobs). The formal fine-mesh T2 cantilever error was 1.158% against the stated Timoshenko-beam comparator. An earlier 3.5% value was only an environment smoke test and is not a formal study result. The other archived benchmark errors and reaction-equilibrium checks verify the numerical setup under those cases, not experimental validation of the trunk.

Mesh convergence used seven three-level studies, two refinement transitions, and four monitored quantities, giving 56 checks (7 × 2 × 4). All 56/56 recorded checks passed. The representative-region von Mises stress tolerance was 10%, and the worst recorded representative-region change was 5.12%. The medium mesh with 7104 C3D8I elements is the frozen formal comparison level; numerical convergence is not a safety criterion.

A fixed-support stress singularity was recorded separately: the base-corner peak stress showed an 11.4% coarse-to-fine change and was excluded from the representative-region stress monitor. Stress comparisons, therefore, use the documented representative region outside the fixed-boundary singularity zone.

### 3.10. Structural Sensitivity and Joint Models

Single-factor structural sensitivity sweeps covered base diameters of 80–120 mm (nominal 100 mm), wall thicknesses of 2, 8, and 16 mm (nominal 4 mm), node-ring thicknesses of 4–8 mm (nominal 6 mm), node-ring lengths of 15–35 mm (nominal 25 mm), and payload conditions represented by LC9–LC11 (0.2, 1.0, and 2.0 kg) plus the LC12–LC13 eccentricity sweep (15 and 45 mm). J1 is a provisional equivalent rotational-spring sensitivity model with *K*_ro*t*_ = 0.1–1.0 N·m/°; it is not a physical joint test or a separate FEA contact model. Its purpose is to identify sensitivity to assumed joint compliance, not to estimate a validated physical joint property or establish a design rule.

J0 is the rigid-joint reference used in the frozen linear FEA: it is the formal baseline FEA condition, an infinite-joint-stiffness idealization, and the basis for the frozen A/C shell comparison. J1 is a provisional equivalent rotational-spring sensitivity model, not a physical joint test; it identifies how A/C displacement ranking depends on assumed joint compliance and produced reversals in four of six specified cases. The J1 ranking reversals do not invalidate the J0 calculation; rather, they show that the J0 ranking is conditional on the rigid-joint idealization and is not robust to all plausible joint-compliance assumptions. J2 is the planned physical torque–rotation calibration and has not been executed. Joint-compliance-dependent A/C design selection is not permitted before J2 calibration, while the reported J0 linear-shell comparison remains a completed conditional computational result.

### 3.11. Run Integrity and Result Provenance

During the structural analysis, a force-couple sign convention error was detected in the first complete run (Run 1) through a physical plausibility check. The eccentric payload moment was applied with an incorrect sign in the force-couple decomposition, causing the symmetric Condition A model to produce asymmetric displacement results among nominally symmetric load cases (LC2 < LC3, contradicting the expected ordering for a symmetric structure under mirror-symmetric loading). The error was traced to a sign inconsistency in the conversion from physical moment convention to nodal force pairs.

After detection of the force-couple sign issue, Run 1 (63 jobs) was superseded as a whole. The currently recoverable Run 1 materials comprise a 63-job summary table, a consolidated run log, and the documented error diagnosis; the complete set of per-job input and solver-output files was not located in the project archive reviewed. The load-application sign convention was corrected, and an 11-check directional test suite was developed; its source code is retained, although a standalone execution record was not located. Run 2 was then executed as the sole source of the manuscript’s structural results. This correction history documents the computational provenance and does not establish that all possible model errors have been excluded.

These surviving materials are retained as part of the computational quality-control and provenance record. 

### 3.12. Planned J2 Physical Joint-Stiffness Calibration

The J1 sensitivity model showed joint rotational stiffness to be influential and produced an A/C displacement-ranking reversal in four of six specified primary cases. This motivated the planned J2 physical torque–rotation calibration. The protocol describes intended measurements of stiffness, hysteresis, backlash, and repeatability; no J2 apparatus data, physical collision tests, or calibration results are reported.

The primary calibration method is direct torque–rotation measurement. In the planned protocol, a single segment interface joint would be mounted in a purpose-designed loading frame. A known force *F* would be applied at a known moment arm *L* from the joint rotation axis to produce a torque *M* = *F* × *L*. The resulting rotation angle *θ* would be measured using a digital inclinometer (resolution 0.01°) and would be verified through visual tracking of fiducial markers. The planned loading protocol would include loading and unloading cycles to characterize hysteresis, repeated measurements at each torque level to quantify repeatability, and measurements at multiple torque levels spanning the expected service range. The primary output is the torque–rotation curve *M*(*θ*), from which tangent stiffness (dM/d*θ* at specified operating points) and secant stiffness (*M*/*θ* over specified ranges) are extracted. Additional characterization includes backlash (rotational free play near zero torque), rate dependence (comparison of quasi-static and dynamic loading), and cycle-dependent stiffness evolution.

J2 physical calibration is required before any joint-stiffness-dependent structural design selection. The current sensitivity analysis shows that assumed joint stiffness can reverse the A/C ranking. Any future rerun should follow a predefined protocol and be supported by new evidence; no J2 test or protocol-defined rerun is reported in this manuscript.

### 3.13. Computational Reproducibility

Computational reproducibility was maintained through frozen configuration snapshots, fixed random seed 20260718 for the expanded LHS, separated inputs and outputs, recorded file hashes, and preserved solver-job status records. Run 2 contains 63 completed formal solver jobs: 26 for Condition A and 37 for Condition C; equivalently, 56 belong to S1 and 7 to S2, with 54 static and 9 linear-buckling jobs. The matrix includes baseline static cases, boundary and parameter-sensitivity cases, bending-stiffness studies, buckling studies, and mesh-refinement jobs; these categories are not collectively relabeled as 13 independent service load cases. Superseded Run 1 is retained only for provenance. Synthetic *J_θ_* trajectories are explicitly excluded from physical evidence claims.

## 4. Results

### 4.1. Protocol-Defined Composite Kinematic Coverage Comparison (H1)

**Figure 4:**
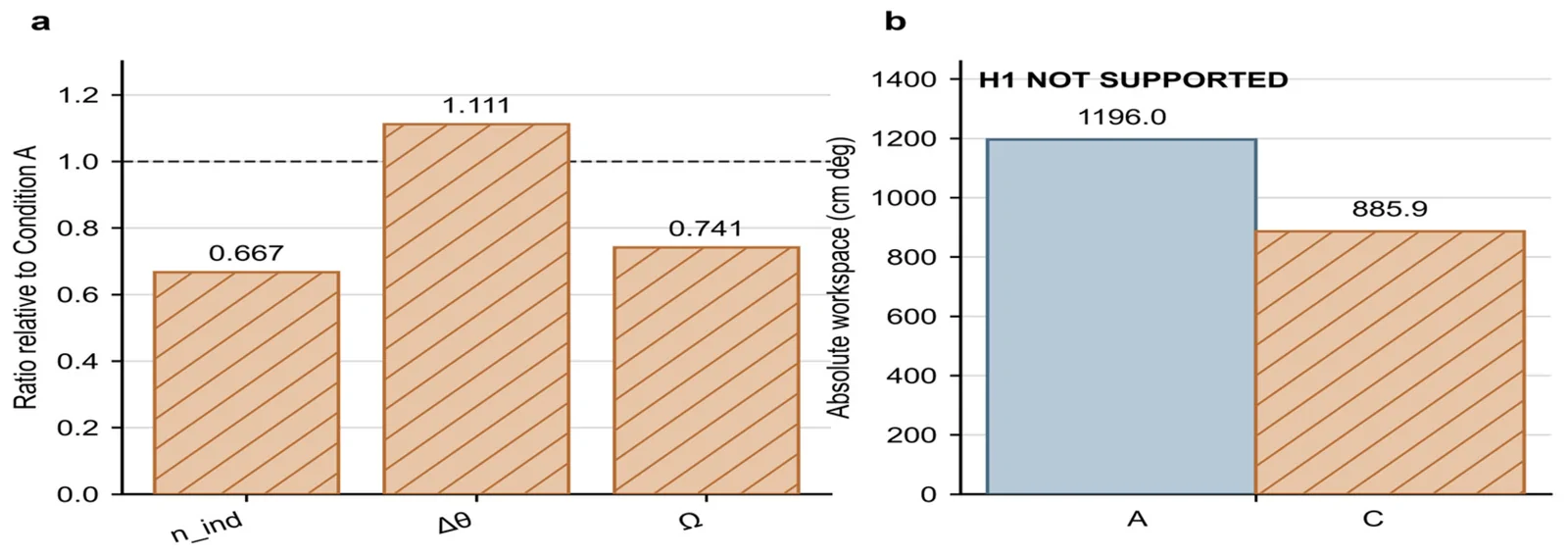
A protocol-defined primary composite kinematic coverage comparison. (**a**) The normalized algebraic component ratios relative to Condition A: *n*_ind,C_/*n*_ind,A_ = 0.667, *Δθ*_C_/*Δθ*_A_ = 1.111, and *Ω*_C_/*Ω*_A_ = 0.741. (**b**) Absolute height-orientation composite coverage: A = 1196.0 cm·° and C = 885.9 cm·°. H1 was not supported under the frozen equal-actuator-budget kinematic conditions.

The primary kinematic comparison is summarized in Figure 4.

The frozen computational H1 test asked whether the MSC-coupled model (C) would provide greater protocol-defined composite kinematic coverage than A under the stated equal-actuator-budget framing. It was not supported: *Ω*_A_ = 1196.0 cm·° and *Ω*_C_ = 885.9 cm·°, giving *Ω*_C_/*Ω*_A_ = 0.741. This is a model-specific comparison of *n*_ind_ = 3 for A and *n*_ind_ = 2 for C, with coupled geometric assumptions. It does not establish a general detrimental effect of morphological coupling or a measured hardware DOF trade-off. Figure 4 reports the primary comparison.

The kinematic comparison, therefore, contrasts *n*_ind_ = 3 for A with *n*_ind_ = 2 for C, while all conditions retain four physical segments and three inter-segment interfaces.

### 4.2. Post Hoc Exploratory Kinematic Analysis

#### 4.2.1. Feasible Coverage and Active-Control Normalization

**Figure 5:**
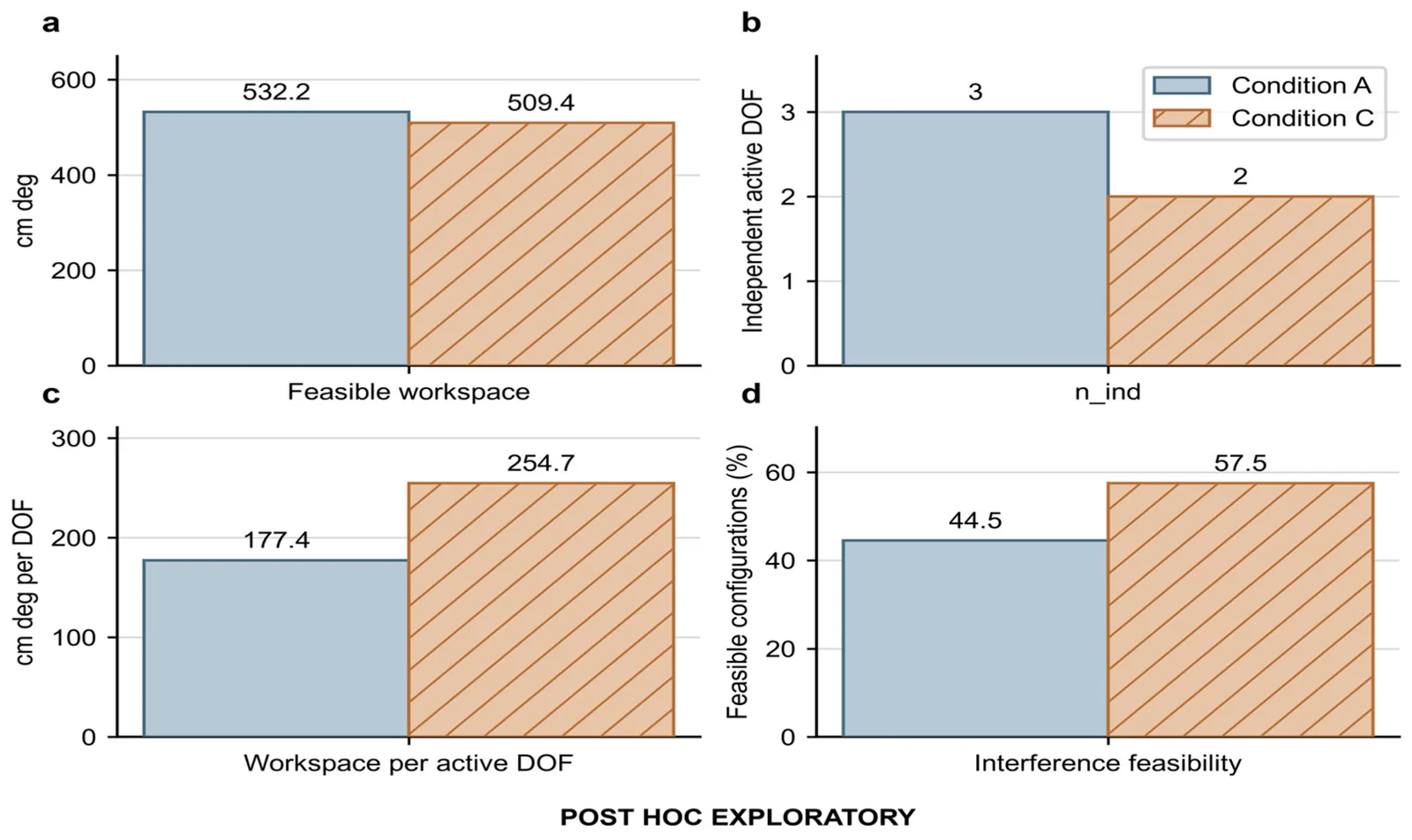
Post hoc exploratory kinematic analysis. (**a**) Feasible composite kinematic coverage. (**b**) The independently active control variables used in the kinematic metric. (**c**) Active-control-variable-normalized coverage and *R*_DOF_ = 1.436. (**d**) Sampled interference-feasibility frequency: A = 44.5% and C = 57.5%. These analyses were defined after the protocol-defined H1 result and are not independent confirmation.

The exploratory feasible-coverage and normalization results are shown in Figure 5.

The post hoc analysis gave *Ω*_feasible, A_ = 532.2 cm·° and *Ω*_feasible,C_ = 509.4 cm·°, so the exploratory feasible-coverage ratio *R*_re*t*en*t*ion_ = 0.957 and the active-control-variable-normalized coverage ratio *R*_DOF_ = 1.436 for the original 200-sample nominal feasibility screen (seed 20260718). 

The reported collision-free values are sampled-configuration frequencies under the frozen model and clearance settings; they are not probabilities of collision-free behavior in a physical robot or venue. In the same nominal screen, the sampled interference-feasibility frequency was 44.5% for A and 57.5% for C.

#### 4.2.2. Parameter Sensitivity and Sampling Stability

**Figure 6:**
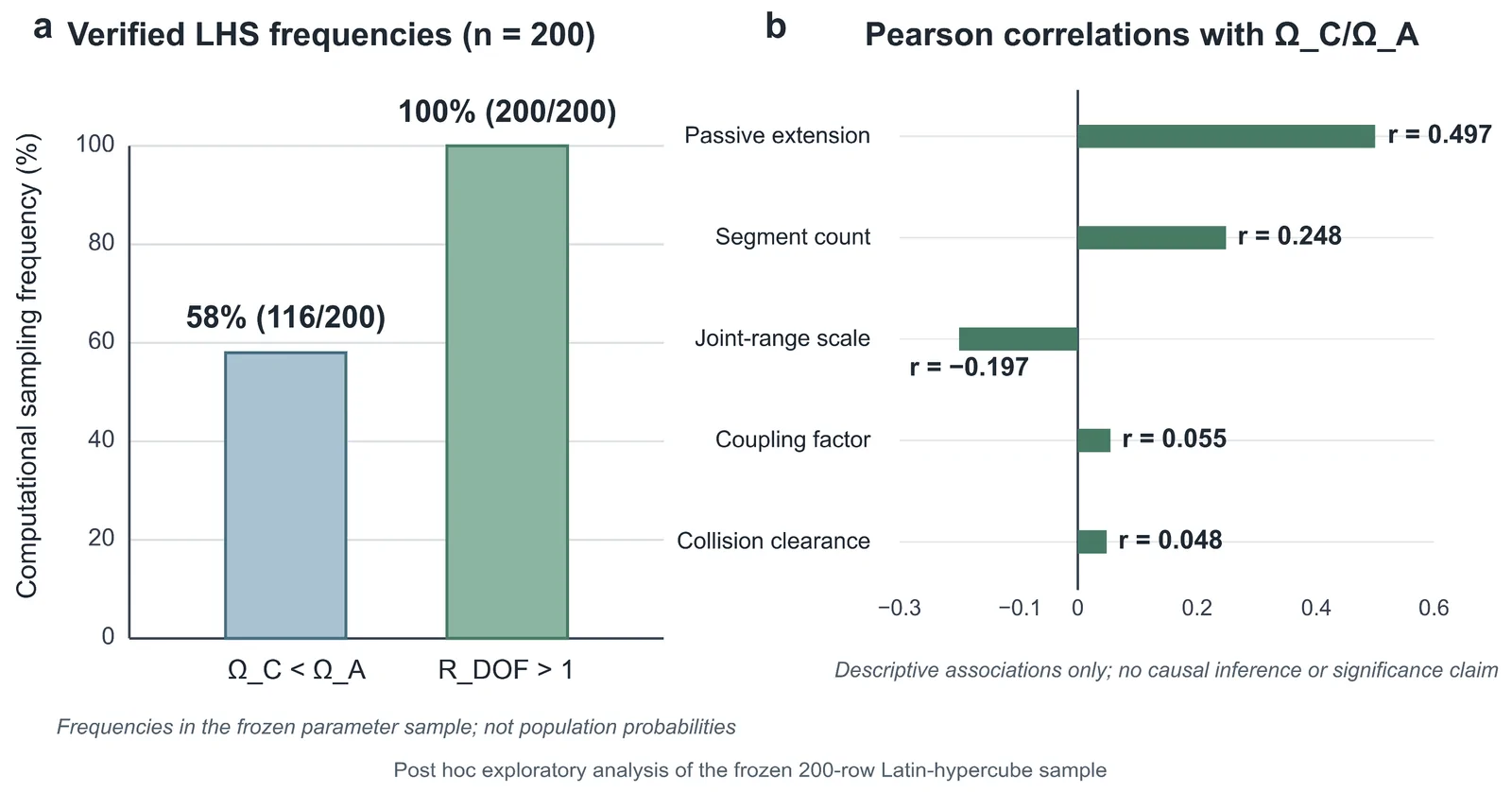
Post hoc kinematic robustness and sensitivity. (**a**) Frequencies within 200 sampled configurations for the two separately defined computational criteria. (**b**) Pearson correlations between the fixed *ΩC*/*ΩA* outcome and the sampled parameters. Sampling frequencies are not population probabilities; correlations are descriptive associations only, with no causal inference or significance tests reported.

In the revision-requested, repeated-seed stability check, the mean values of *R*_re*t*en*t*ion_ were 0.996, 0.992, 0.989, 0.984, and 0.976 at *N* = 50, 100, 200, 500, and 1000, respectively; the mean values of *R*_DOF_ were 1.494, 1.487, 1.484, 1.475, and 1.464. At *N* = 1000, the empirical 2.5–97.5th percentile ranges were 0.939–1.018 for *R*_re*t*en*t*ion_ and 1.408–1.527 for *R*_DOF_. These are descriptive sample-size and seed-stability summaries, not a replacement for the original 0.957 estimate, a confirmatory replication, an independent validation, or statistically proven convergence. Figure 6 presents the corresponding sampled frequencies and Pearson correlations.

In the 200-sample expanded post hoc Latin-hypercube analysis, 116/200 samples (58%) had *Ω*C < *Ω*A, and 200/200 (100%) had RDOF > 1. The 58% result is only a slight majority and is parameter-dependent; neither computational sampling frequency is a population probability nor an independent validation result. The repeated-seed check varied feasibility sample size at fixed nominal geometry and, therefore, addresses numerical sampling stability only; it does not validate the model or prove convergence. Pearson correlation coefficients from the frozen 200-sample analysis are descriptive exploratory associations (Figure 6), not causal effects.

All analyses in Section 4.2 are exploratory and hypothesis-generating; none is used to reinterpret or confirm the unsupported primary H1 result.

### 4.3. Solver Validation and Mesh Convergence

**Figure 7:**
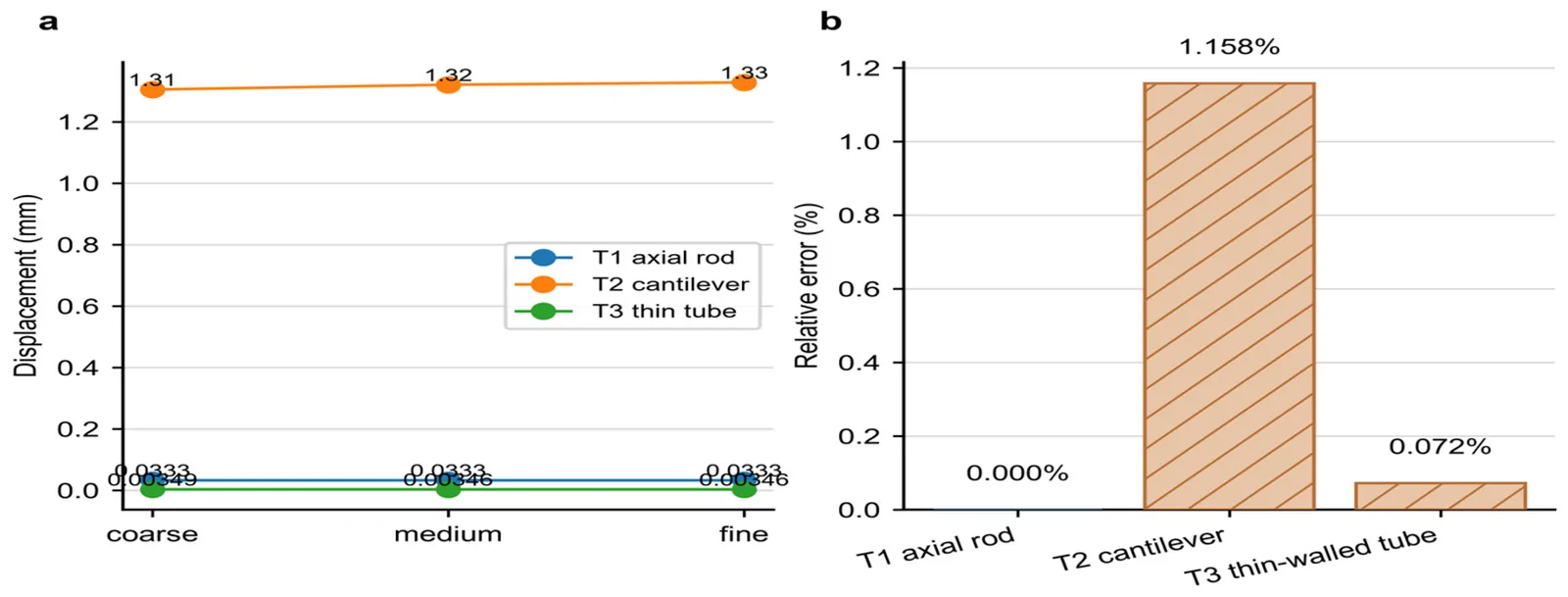
The solver validation. (**a**) Analytical-versus-FEA displacement convergence for the T1 axial rod, T2 cantilever beam, and T3 thin-walled tube. (**b**) Fine-mesh relative errors; the formal T2 C3D8I error is 1.158% against Timoshenko-beam theory. These are solver validation data, not study performance results.

**Figure 8:**
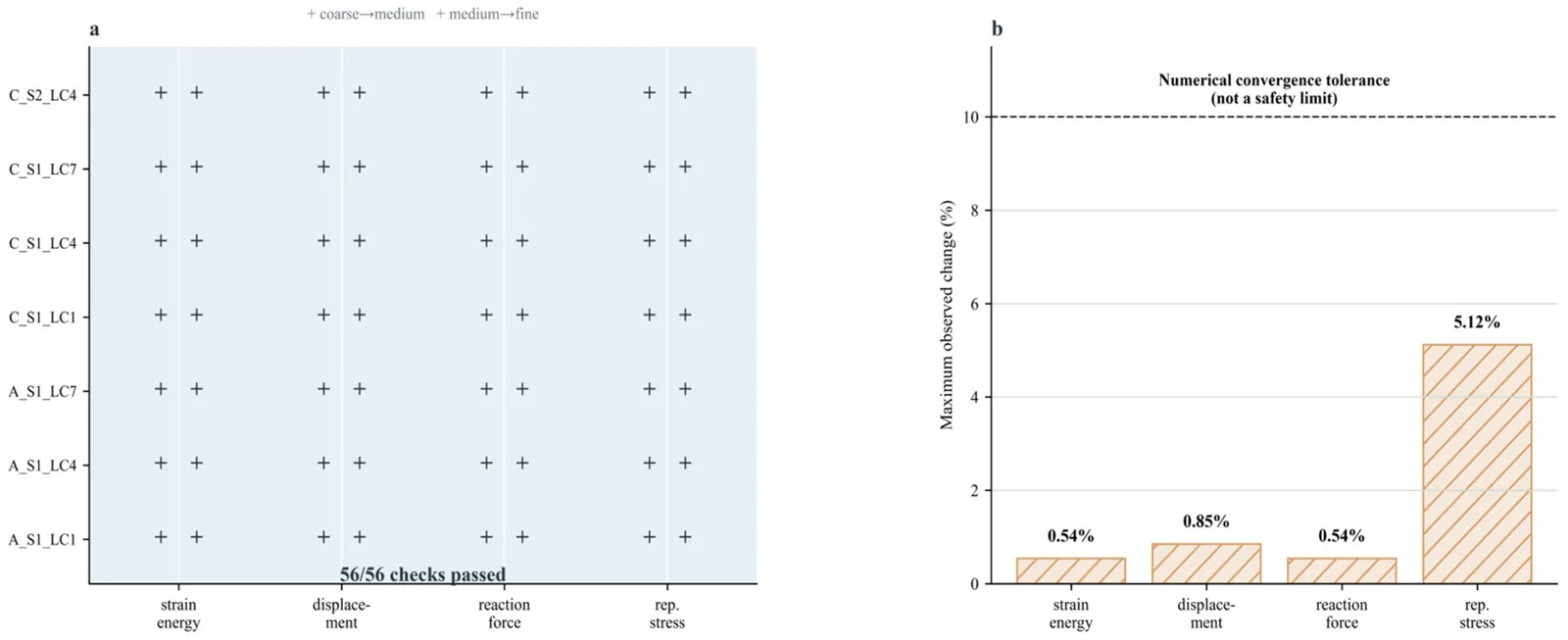
The numerical mesh-convergence assessment. (**a**) Coarse-to-medium and medium-to-fine checks across seven studies and four monitored quantities; all 56 checks passed. (**b**) The maximum observed change by quantity with pre-specified numerical convergence tolerances. The 10% line is a numerical convergence tolerance, not a safety limit. The displayed values are computational convergence results only.

Solver benchmarking and mesh convergence are summarized in Figure 7 and Figure 8, respectively.

The formal solver validation used three analytical benchmark problems at three mesh densities. The formal fine-mesh T2 error was 1.158% against the stated Timoshenko-beam comparator; 3.5% refers only to an early environment smoke test and is not a reported benchmark result. The trunk mesh protocol comprised 56 = 7 × 2 × 4 checks, all of which passed; the representative-region stress tolerance was 10%, and the worst observed change was 5.12%. Figure 7 and Figure 8 show the benchmark and mesh-convergence records. These checks establish numerical consistency under the documented setup, not physical validation.

### 4.4. Structural Mass–Stiffness Comparison (H2)

**Figure 9:**
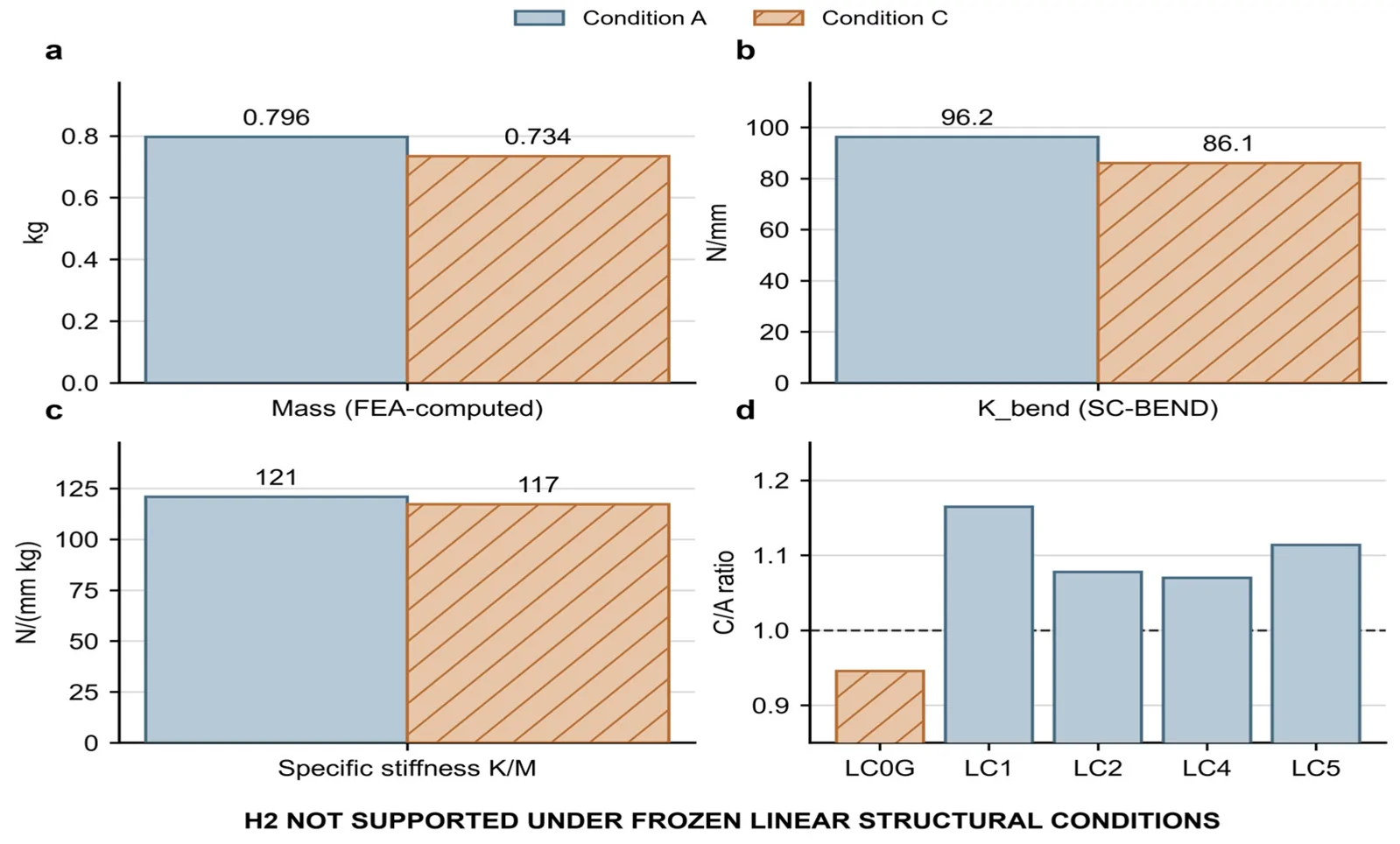
Structural comparison under S1 (equal external envelope and payload). (**a**) Mass. (**b**) The bending stiffness. (**c**) The mass-normalized stiffness. (**d**) The C/A interface-displacement ratio by the load case. H2 was not supported under the rigid-joint (J0), linear-elastic 500 mm subsystem comparison; J1 sensitivity is reported separately. LC0G is displayed as a neutral exception and does not alter the overall H2 status.

**Figure 10:**
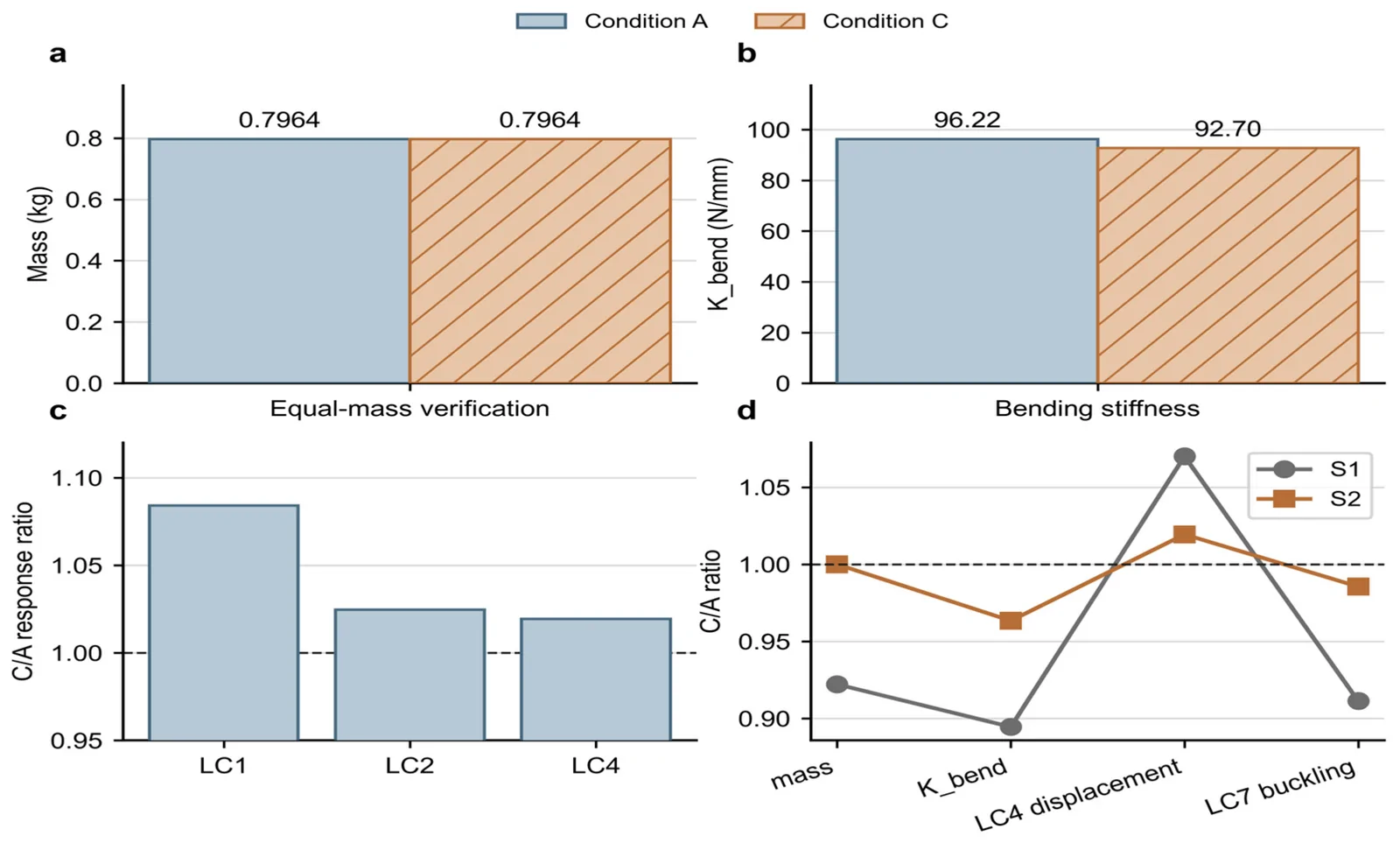
Equal-mass structural comparison under S2. (**a**) Equal-mass verification after wall-thickness scaling *s* = 1.0883. (**b**) The bending stiffness. (**c**) The selected verified C/A response ratios. (**d**) S1-versus-S2 C/A gap comparison for mass, bending stiffness, LC4 displacement, and LC7 buckling. The S2 bending-stiffness gap remains approximately 3.7%; H2 remains not supported under the rigid-joint (J0), linear-elastic 500 mm subsystem comparison.

The S1 and S2 structural comparisons are shown in Figure 9 and Figure 10, respectively.

The frozen computational H2 test asked whether the MSC-coupled tapered model (C) would show superior mass-specific structural efficiency to A. H2 was not supported under the rigid-joint (J0), linear-elastic 500 mm subsystem comparison. Under S1, C had approximately 7.8% lower model mass, but 10.5% lower calculated bending stiffness and 3.0% lower calculated mass-specific stiffness. Under S2, the stiffness gap narrowed, but A remained ahead on the reported primary metrics. These outputs are conditional model results, not a universal verdict on a bamboo-inspired structure or a physical design ranking. These comparisons characterize the shell/subsystem response under J0 and should not be interpreted as a joint-compliance-independent ranking of complete physical assemblies. Figure 9 and Figure 10 and Table 1 report the two structural comparison regimes.

### 4.5. Joint-Stiffness and Structural-Parameter Sensitivity

**Figure 11:**
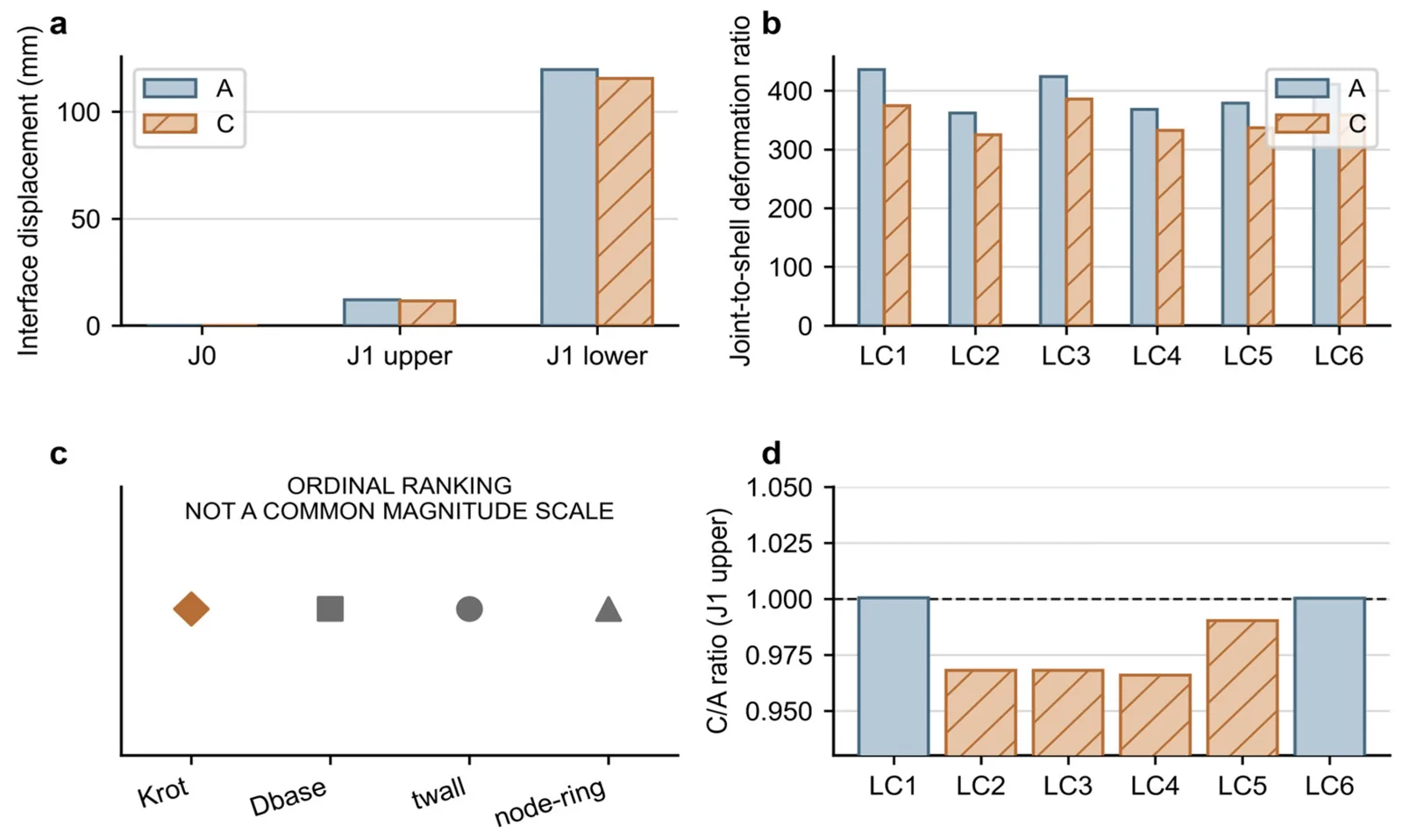
Joint-stiffness sensitivity under the J1 provisional equivalent rotational-spring model. (**a**) J0, J1_upper_, and J1_lower_ interface displacement at LC4. (**b**) The joint-to-shell deformation ratios across six load cases. (**c**) Ordinal structural-parameter influence ranking, not a common magnitude scale. (**d**) The C/A ratios across six J1_upper_ cases. J1 is not J2 physical calibration; the J0 structural ranking is conditional, and joint-compliance-independent hardware ranking remains unresolved.

Figure 11 shows the provisional joint-stiffness sensitivity results.

Within the provisional J1 equivalent rotational-spring range of 0.1–1.0 N·m/°, joint rotational compliance showed the largest modeled contribution to system displacement relative to the shell parameters examined. Four of six specified primary cases showed an A/C displacement-ranking reversal relative to the J0 rigid-joint reference. This is a provisional equivalent-spring result based on assumed stiffness, not measured torque–rotation data. The J1 ranking reversals do not invalidate the J0 calculation; rather, they show that the J0 ranking is conditional on rigid-joint idealization and is not robust to all plausible joint-compliance assumptions. The planned J2 physical calibration has not been executed, so no joint-compliance-dependent A/C design selection is made.

Table 2 consolidates the distinction between hypotheses not supported by the reported computational comparison and hypotheses not yet tested.

## 5. Discussion

### 5.1. Interpretation of the Unsupported Composite-Coverage Hypothesis

The remaining results are interpreted as frozen computational outputs under the stated model assumptions; no external preregistration, deployment, prototype test, or user study is claimed.

The nominal coupling factor of 0.3 is one frozen engineering-model setting. It should not be interpreted as sufficiently strong, optimal, or physically calibrated coupling. Within the current algebraic model, the coverage loss associated with fewer independent kinematic combinations is not recovered by the modeled passive extension at this setting; this is a model-dependent trade-off, rather than a physical design rule.

Within the current algebraic model, the crossover occurs near a coupling-factor value of approximately 0.10 under the other frozen assumptions. This is a model-derived crossover, not a validated physical design threshold. The result should not be used to specify a coupling factor for a physical robot.

The H1 negative result contributes a methodological point: biomechanically inspired design features should not be presumed to confer performance advantages. They must be evaluated through controlled, like-for-like comparison against a conventional baseline under explicitly stated fairness conditions. The A/C comparison regime adopted here—the equal actuator count and the equal segment count, differing only in whether segments are independently controlled (A) or mechanically coupled (C)—provides a template for such evaluation.

### 5.2. Morphological Coupling and Active-DOF Efficiency

Following the H1 negative result, a set of post hoc exploratory metrics was defined to examine whether the MSC-coupled configuration might offer advantages in dimensions other than absolute composite kinematic coverage. These metrics must be clearly distinguished from the protocol-defined H1 test: they were formulated after the negative H1 result was known, computed on the same nominal model, and carry the mandatory label post hoc exploratory. They do not constitute a redefinition or rescue of H1. The repeated-seed analysis addresses sample-size and seed stability of these computational screening quantities only; it does not convert them into confirmatory evidence.

The exploratory feasible-coverage ratio. Despite its 25.9% smaller absolute composite kinematic coverage, Condition C retained 95.7% of Condition A’s feasible composite coverage (*Ω*_feasible,C_/*Ω*_feasible,A_ = 0.957) in the original nominal 200-sample screen. This is an exploratory feasible-coverage ratio, not performance retention. It suggests that a portion of the coverage lost through DOF reduction consists of configurations that are kinematically reachable but classified as infeasible by the provisional sampled interference screen. The MSC-coupled geometry may, therefore, merit follow-up study, but this interpretation remains model- and definition-dependent.

The active-control-variable-normalized coverage ratio. The original post hoc model-derived value was *R*_DOF_ = 1.436. This ratio exceeds 1.0 in 200/200 samples (100%) of the expanded parameter LHS, but the result is definition-dependent because the normalization explicitly uses *n*_ind_. Because *n*_ind_ appears explicitly in the normalization, *R*_DOF_ is definition-dependent and cannot by itself establish an intrinsic morphological or structural efficiency advantage. It is, therefore, a hypothesis-generating normalized comparison, not evidence that the coupled morphology is more efficient in an intrinsic or hardware-realized sense.

Pearson correlations across the 200-row parameter sample showed the largest positive descriptive association with passive joint extension (r = 0.497), whereas the coupling factor showed a weak association (r = 0.055). These are model-dependent trends rather than validated design rules; they identify candidate variables for future physical calibration rather than prescribing physical performance.

H6 was formulated prospectively for future independent testing after the post hoc exploratory finding; it was not tested with independent data.

### 5.3. Interpretation of the Unsupported Structural Hypothesis

H2 is reported only as a frozen computational comparison under the rigid-joint (J0), linear-elastic 500 mm subsystem model. It was not supported in the reported model and should not be interpreted as a prototype result, a complete-assembly ranking, or a general-design conclusion. J1 sensitivity shows that assumed joint compliance can alter A/C displacement ranking, and J2 physical calibration has not been executed.

The structural comparison produced internally consistent J0 model results across two regimes. Under S1 (equal envelope and equal load), Condition C showed a model-calculated 7.8% mass reduction (0.7344 vs. 0.7964 kg), attributable to the tapered geometry and hollow cross-section inherited from the bamboo-inspired parameterization. However, the bending stiffness decreased by 10.5% (*K*_bend_: 86.08 vs. 96.22 N/mm), and the mass-specific stiffness decreased by 3.0% (*K*/*M*: 117.2 vs. 120.8 N/mm·kg). Under S2 (equal mass, achieved by scaling C wall thickness), the stiffness gap narrowed, but A remained ahead on the reported metric. These values characterize the stated J0 shell/subsystem model and do not establish a joint-independent hardware ranking.

Two narrow exceptions are noted. Under self-weight-only loading (LC0G), Condition C exhibited smaller tip displacement (C/A = 0.946), consistent with its lower self-weight reducing the gravitational load. Under forward-leaning buckling (LC8), Condition C showed a marginally higher first eigenvalue (C/A = 1.009), and the difference (+0.9%) exceeds the reported medium-mesh Richardson error estimate (≤0.6%); it is not interpreted as evidence of a general buckling advantage. These exceptions do not alter the overall direction of the comparison and do not constitute support for H2.

Within the J0 comparison, the direction of the S1 and S2 results is consistent, but this does not resolve the effect of compliant joints or establish generality beyond the modeled subsystem. The bamboo-inspired tapered geometry reduces mass, but the stiffness loss exceeds the mass savings in the tested configuration. The node-ring features contributed less than 2.5% to any structural metric under linear-elastic conditions; they are geometrically present but structurally marginal in this regime.

Several factors limit the generality of this finding. The analysis used small-deformation, linear-elastic material behavior (PLA); it does not represent TPU nonlinear compliance, large-deflection behavior, joint contact or friction, backlash, tendon/cable compliance, human contact, dynamic impact, or assembled-robot collision safety. The 500 mm domain is a controlled structural subsystem and is not a geometrically scaled representation of the approximately 1300 mm conceptual whole-product envelope; no direct full-height extrapolation has been made. J1 remains a provisional equivalent-spring sensitivity model, and J2 physical torque–rotation calibration has not been executed. H2 is, therefore, characterized as not supported under the frozen J0 linear-elastic subsystem conditions, not as a universal falsification of bamboo-inspired structural design.

### 5.4. Joint Stiffness as a Key Unresolved Variable

Within the provisional J1 assumptions, the modeled joint contribution to tip displacement exceeded the elastic beam-bending contribution across the tested range. This is a model-dependent sensitivity trend based on assumed stiffness, not a validated physical design rule or a claim about a completed assembly.

Four of the six primary load cases (LC2–LC5) exhibited a reversal in the A/C displacement ranking under J1 conditions. The mechanism is straightforward: Condition C’s lower self-weight reduces the gravitational component of the joint moment, partially compensating for its lower structural stiffness. This reversal is a provisional sensitivity result—it is computed from analytical joint stiffness estimates, not from physically measured moment–rotation curves—but it indicates that the linear-model ranking is sensitive to the unresolved J1 assumption.

Within the provisional J1 sweep, the parameter sensitivity ordering placed joint rotational stiffness (*K*_ro*t*_) ahead of the shell parameters examined, followed by base diameter; node-ring parameters ranked lower within this specific model. This ordering is descriptive and model-dependent, not a universal design rule. It supports prioritizing future joint characterization rather than selecting a final geometry from the current sensitivity result.

The J2 physical calibration protocol, designed but not yet executed, will measure the moment–rotation relationship (including loading/unloading hysteresis, backlash, and secant stiffness) of the PLA segment connectors. Until J2 is completed, the structural comparison between Condition A and Condition C remains provisional. No design selection between A and C should be made on the basis of the current structural data alone.

### 5.5. Implications for Bamboo-Inspired Morphological Design

The combined kinematic and structural results offer methodological lessons for the practice of bamboo-inspired morphological design in robotics, beyond the specific A/C comparison reported here.

Separating resemblance from performance. The bamboo-inspired features—node rings, internode hollowness, taper gradient, and segment modularity—were translated into specific, independently varied design variables (*t*_node_, *D*_i_/*D*_o_, *t*_aperra*t*io_, *s*_egmen*t*coun*t*_, and *c*_ouplingfac*t*or_). This parameterization enables a distinction that is often blurred in the biomimetic design literature: cultural or aesthetic resemblance to a biological prototype is not evidence of engineering performance. The H1 and H2 negative results demonstrate that morphological similarity does not guarantee functional superiority, even when the biological source organism (bamboo) exhibits the target function (structural efficiency) in its native context. The translation from biological mechanism to engineering variable must be evaluated at the level of measurable function, not at the level of feature count or visual analogy. This separation is also important where cultural semantics and sensory evaluation are studied: those outcomes require their own evaluation design, rather than being inferred from structural calculation [15,16,17,18].

Mechanism-based translation. This study uses a four-level pathway: biological mechanism identification → engineering abstraction (MSC framework) → computational parameterization → comparative evaluation. This pathway keeps cultural inspiration, biological interpretation, engineering implementation, and model outputs distinct. Related work on traditional-craft-mediated biomimetic translation and hierarchical evaluation similarly treats translation from analogue to engineering variable as reviewable rather than self-validating [13,14].

Negative results as design knowledge. The H1 and H2 negative results contribute to a cumulative understanding of bamboo-inspired structural design. Knowing that a specific parameterization of MSC coupling (taper = 0.15, coupling = 0.3, and hollow ratio = 0.92 at Dbase = 100 mm) does not, under frozen comparison conditions, outperform a conventional rigid design on composite kinematic coverage or structural stiffness is itself a design-relevant finding. It constrains the space of promising configurations and directs attention toward variables that require physical calibration, such as joint stiffness, base diameter, passive extension, and contact behavior, rather than treating any one variable as a validated universal driver.

The A/C contrast is a protocol-defined comparison with coupled differences in passive coupling, taper geometry, and active-control-variable count. It does not isolate a single independent variable or establish causality beyond the stated model.

These methodological points are offered as transferable principles, not as a claim of a validated design framework. They remain bounded by the computational nature of the present evidence and the specific geometry, material, and load conditions investigated.

### 5.6. Regional and Cultural Design Implications for Xiang–Gan Museums

The present study is bounded to the Xiang–Gan red-culture museum context. This regional constraint is integral to the research design, not a secondary limitation. Its relevance lies in examining how visitor-facing service needs and region-specific cultural references might inform robot morphology within the same design problem, rather than treating the robot as a context-free mechanical structure.

The named Xiang–Gan heritage venues provide a contextual design frame. Their operational conditions have not been measured in this study, and future fieldwork is needed before venue-specific requirements are asserted.

Cultural morphology. In this study, Jinggang bamboo imagery is used as a contextual cultural reference associated in regional revolutionary narratives with resilience, disciplined growth, and historical memory. The MSC design’s segmented, tapered, and hollow visual profile draws on this regional cultural imagery, rather than on an asserted local bamboo species identity. Whether museum visitors and staff perceive this morphological reference as culturally meaningful has not been empirically evaluated. H5, which posits that bamboo-inspired cultural morphology enhances perceived cultural identity, therefore, remains untested. The present study provides the morphological parameterization and the comparative design conditions (A, B, C) that could support a future cultural-perception study, but it does not provide evidence for or against the cultural-identity claim itself.

Distinguishing cultural from biological evidence. This study maintains a strict separation between two registers of bamboo reference: (a) Jinggang bamboo as a cultural symbol—a regional, historically contextualized cultural imagery that informs the design’s visual language and its potential resonance with Xiang–Gan audiences; and (b) generic bamboo biomechanics as a biological knowledge source—node–internode organization, hollow tapered geometry, and graded material properties documented in the broader biomimetic engineering literature. Claims in register (a) require cultural perception evidence (not yet collected); claims in register (b) require biomechanical measurement or validated computational modeling (the present computational results are provisional in this regard). The two registers are not interchangeable, and evidence from one does not satisfy the evidential requirements of the other.

Regional constraint enforcement. All conclusions are explicitly bounded to the Xiang–Gan red-culture museum context. No claim is made about the applicability of these findings to museums in other Chinese regions, to general heritage sites, or to international museum contexts. This constraint applies to the service scenario descriptions, the cultural morphology claims, and the design implications drawn from the computational comparison.

### 5.7. Comparison with Previous Biomimetic and Robotic Studies

The present study occupies a specific intersection—bamboo-inspired morphological design, service-robot trunk structures, and revolutionary heritage museum applications—that limits the scope of direct comparison with prior work. Relevant comparisons are organized by research strand.

Bamboo-inspired exoskeletons and structural elements. BiEXO demonstrated bamboo-inspired hollow tubular links in a lightweight wearable exoskeleton [11]. The present study asks a different, computational question: can passive morphology–structure coupling reduce actuation while retaining task-relevant composite kinematic coverage? It does not claim superiority over the wearable application.

Morphological computation and embodied intelligence. The present MSC formulation is a narrow computational design hypothesis informed by the morphological computation and co-design literature [29,30,31,32]. It does not demonstrate dynamic adaptation, perception-action closure, or embodied intelligence.

Cultural references in this manuscript motivate a future design-evaluation question; no site-specific deployment, visitor response, or cultural-perception outcome is reported.

Comparison limitations. All comparisons in this section are interpretive and qualitative; no quantitative meta-analysis or formal benchmarking against prior studies has been performed. The comparisons use the cited literature only as contextual reference, and no claim of systematic superiority over previous work is made.

### 5.8. Evidence Scope and Claim Boundaries

The present work is a computational design study with deliberately limited evidential scope. Engineering validation, venue-task research, and cultural-semantic evaluation require separate future studies using appropriate physical, field, or user data.

Accordingly, the results should be interpreted as computational design evidence rather than as a deployment study or product-validation report.

MSC coupling is treated here as a computational design hypothesis involving passive mechanical constraint and reduced independent control variables, not as validated embodied intelligence. Future physical calibration, nonlinear/contact analysis, prototyping, control testing, and museum evaluation are distinct prospective stages; none has been completed.

### 5.9. Priorities for Future Validation

Priority 1 is J2 physical torque–rotation calibration of the segment connector, including stiffness, loading/unloading behavior, backlash, and repeatability. Until those measurements exist, J1 remains a provisional sensitivity model, and no joint-compliance-dependent A/C design selection is warranted.

Priority 2 is a prospectively specified structural re-analysis after the J2 data become available. Its cases, acceptance criteria, and interpretation must be approved before execution; no such rerun is reported here.

Priority 3 is material characterization if a future nonlinear/contact study is pursued. The present linear PLA model is not suitable for claims about compliant TPU contact behavior.

Priority 4 is future nonlinear/contact analysis for H3 after joint and material prerequisites are met. No nonlinear contact result or physical contact-force evidence is reported in this manuscript.

Priority 5: Minimum-function physical prototype. A physical prototype of the 500 mm trunk test section, fabricated using PLA with instrumented joints, would enable dynamic testing of H4-A (active control reduction after disturbance) and provide empirical data on manufacturing tolerances, assembly repeatability, and joint degradation under cyclic loading—all of which are absent from the current computational models.

Priority 6: Venue-specific task validation. Operational testing in the three core Xiang–Gan venues, even at the level of task simulation using venue-derived spatial constraints and service scenarios, would substantially strengthen the ecological validity of the composite kinematic coverage and sampled interference-feasibility comparisons. Such testing requires institutional cooperation and must follow heritage site access protocols.

Priority 7: Cultural perception and user studies. H5 (cultural identity enhancement) requires a separate methodological approach—user perception studies with Xiang–Gan regional participants, using validated cultural identity measurement instruments and controlled comparison of bamboo-inspired versus conventional trunk morphologies. This work falls outside the computational and engineering scope of the present study and would benefit from collaboration with cultural-heritage and museum studies researchers. Existing cultural–craft sensory and perception studies provide relevant examples of separate user-facing evaluation pipelines, but they do not validate the present robot concept [15,17,18].

All future work descriptions are prospective plans, not reports of ongoing or completed activities. No institutional approval, funding commitment, or timeline is implied for any of the listed priorities.

### 5.10. Evidence-Constrained Conceptual Design Synthesis

#### 5.10.1. The Design Constraints Derived from the Computational Results

The conceptual synthesis begins from constraints and presents a candidate conceptual configuration for future validation, not an optimized or selected final design. Under the frozen equal-actuator-budget comparison, the MSC-coupled Condition C did not expand absolute composite kinematic coverage: *Ω*_C_ was 885.9 cm·° compared with *Ω*_A_ of 1196.0 cm·°, yielding *Ω*_C_/*Ω*_A_ = 0.741. The resulting architecture, therefore, does not present a larger reachable envelope as its advantage. The retained post hoc observation is narrower: the coupled condition retained 509.4 cm·° of feasible composite coverage relative to 532.2 cm·° for A (*R*_re*t*en*t*ion_ = 0.957), while the active-control-variable-normalized coverage ratio was *R*_DOF_ = 1.436. These exploratory quantities motivate future design questions about task-relevant configuration efficiency and physical contact calibration; they do not validate a service benefit or justify a claim of superior adaptive behavior.

The frozen rigid-joint J0, linear-elastic 500 mm subsystem comparison imposes a second constraint. Condition C is approximately 7.8% lighter, but its bending stiffness was approximately 10.5% lower and its mass-normalized stiffness approximately 3.0% lower under the stated conditions; H2 was not supported under this conditional comparison. The synthesis does not interpret reduced mass as a structural-efficiency gain. Instead, it retains a compact tapered shell as a candidate conceptual variable whose wall, diameter, and node-ring parameters must be reconsidered alongside joint behavior. No joint-compliance-independent hardware ranking or design selection is claimed.

#### 5.10.2. Morphological and Product Architecture

The candidate conceptual configuration is a modern wheeled museum service-robot architecture, derived from bamboo structural logic rather than styled as a bamboo stalk. A compact mobile base supports four vertically articulated trunk modules with a controlled lower-to-upper taper and three visible node-ring interfaces. Two interfaces are designated as active concept interfaces and one as a passive-coupled concept interface in correspondence with the frozen A/B/C abstraction; this designation is architectural and does not specify an actuator, transmission, tendon mechanism, or degree-of-freedom count beyond the established comparison model. An upper interaction display and sensor band provide a service-robot reading, while maintenance seams, a provision for future cable routing, and a J2 calibration/removal access are included to preserve the dependency of later work on measurable joint behavior. The current Condition C does not specify or numerically model a validated tendon transmission.

**Figure 12:**
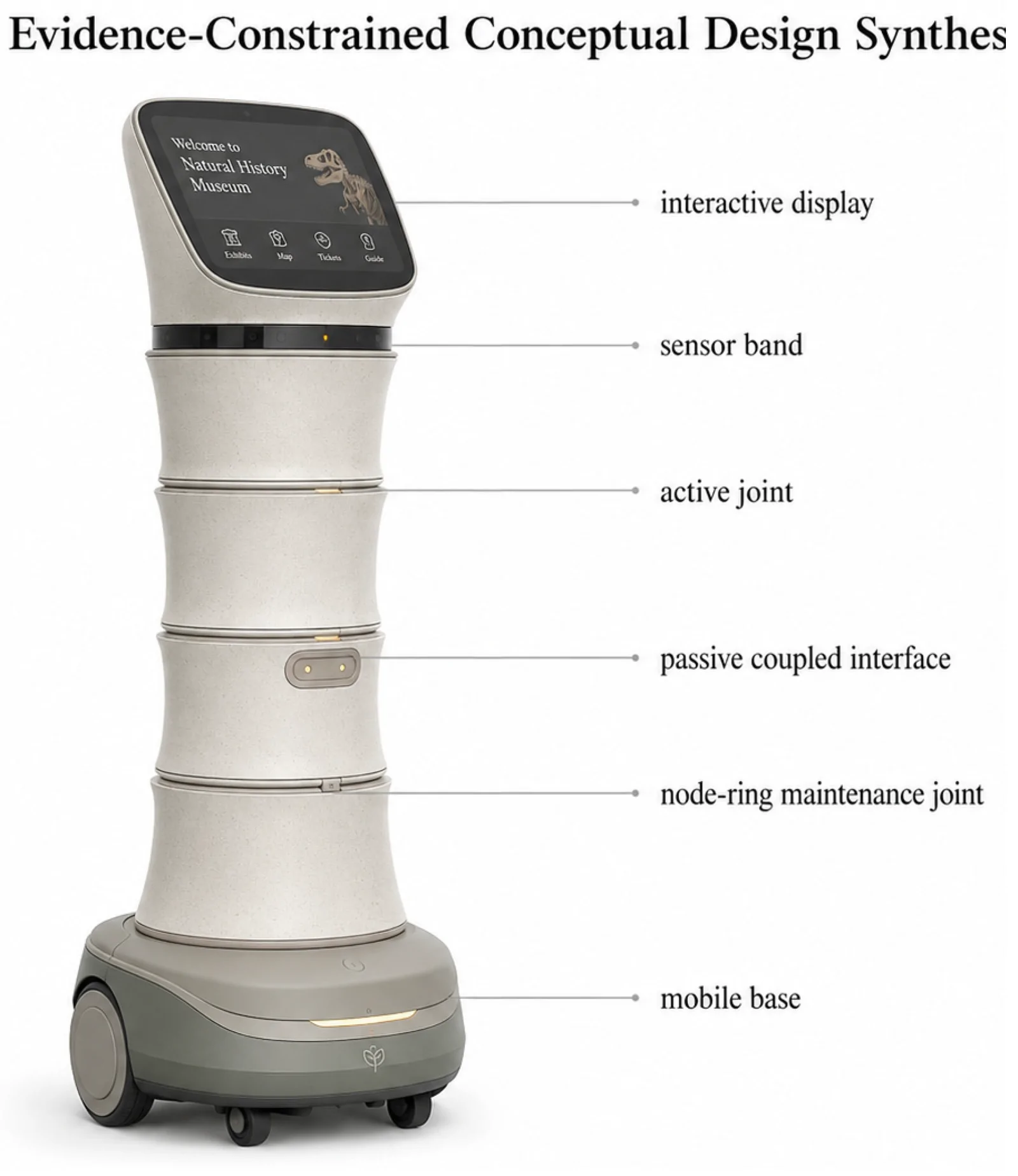
A candidate conceptual configuration for future validation: evidence-constrained design synthesis of the bamboo-inspired museum service robot. The full-product concept translates frozen computational outcomes into design constraints rather than asserting that Condition C is superior to A. The four-module and three-interface architecture includes conceptual maintenance and calibration access because joint stiffness remains subject to the planned J2 physical calibration. This is not an optimized or experimentally validated design and is not a physical prototype.

**Figure 13:**
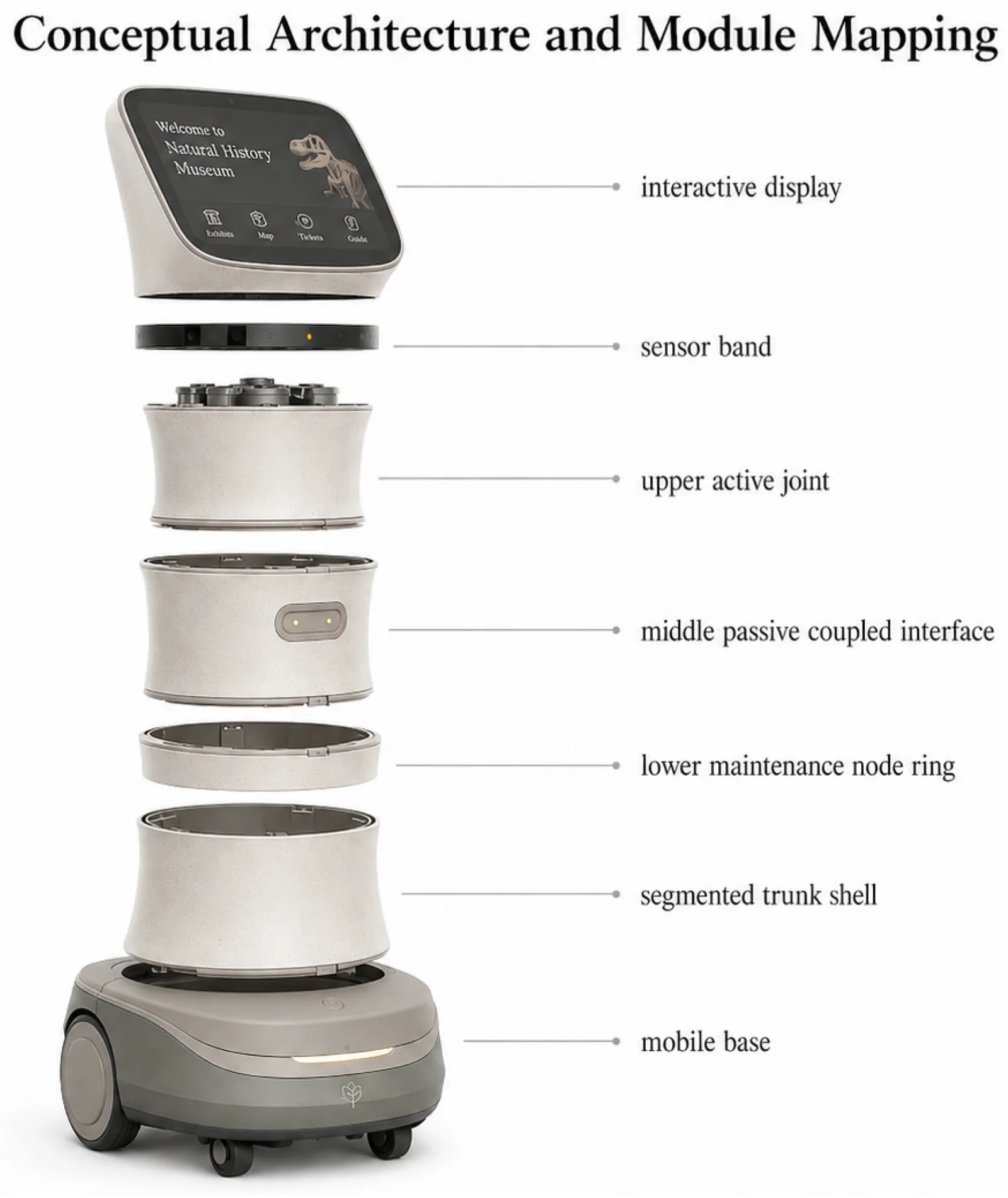
A candidate conceptual architecture for future validation, showing the mapping between the design modules and computational variables. The diagram is not a manufacturing drawing or an experimentally validated configuration. The part dimensions, interfaces, and joint parameters remain subject to revision after J2 calibration and subsequent structural validation.

The approximately 1300 mm concept shown in the design synthesis is a product-level visualization constrained by the computational findings; it is not a structural extrapolation of the 500 mm FEA domain. The 500 mm structural-analysis domain remains a controlled subsystem model rather than a geometrically scaled representation of the conceptual whole-product envelope; no manufacturing dimension or performance inference is drawn from the visual scale mapping. Figure 12 and Figure 13 illustrate the candidate configuration and its conceptual architecture, respectively.

#### 5.10.3. Intended Functional Configurations

**Figure 14:**
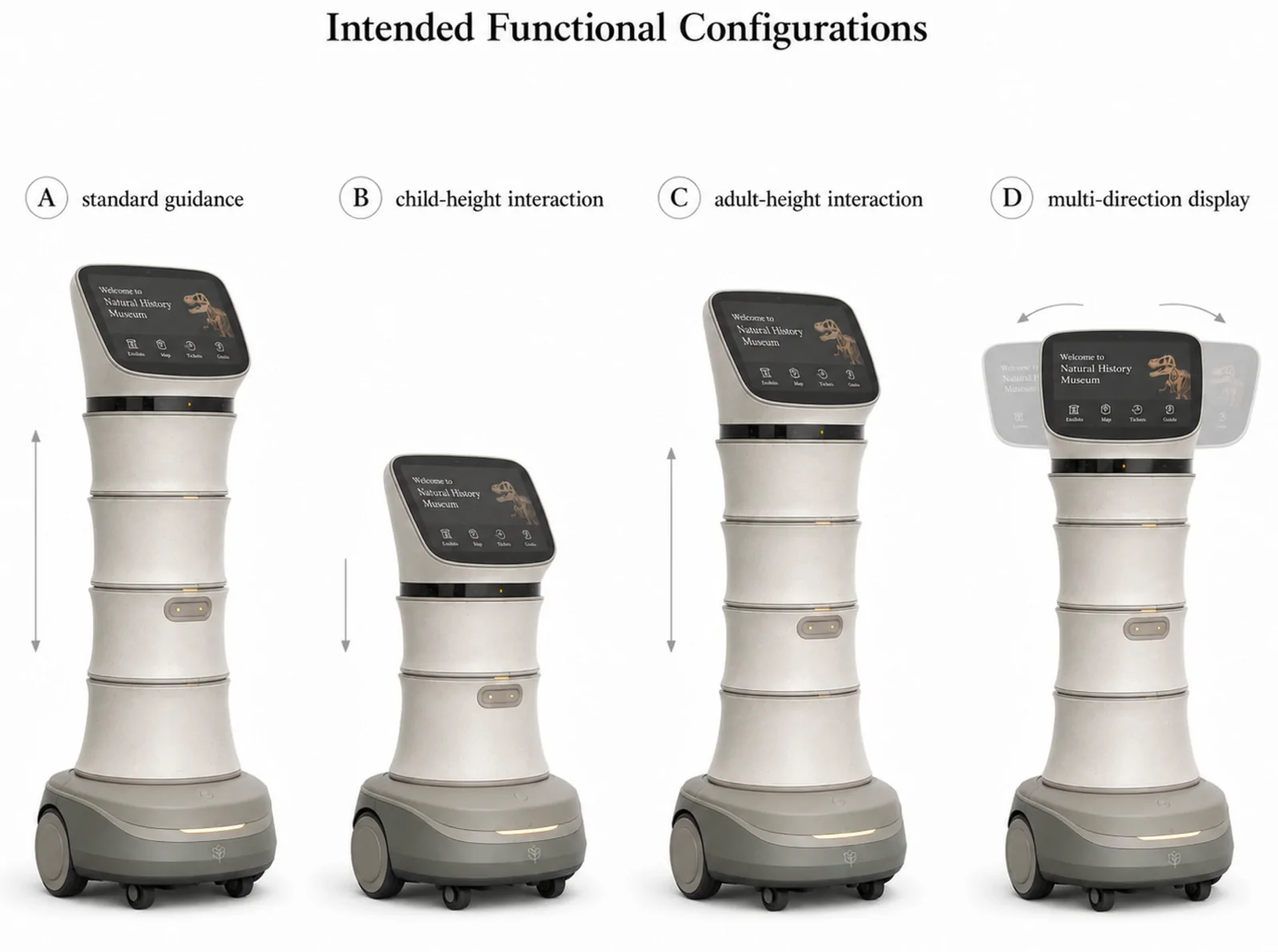
Candidate intended functional configurations of the conceptual robot trunk for future validation. These are conceptual arrangements, not tested modes, optimized configurations, or validated working states.

Four intended functional configurations are shown as bounded candidate conceptual arrangements for future validation: standard interpretation, child-height interaction, standing-adult interaction, and multi-directional interface orientation. They are not tested modes or validated working states. The drawings retain the same base and four-module architecture and use only modest display and module offsets, rather than unverified large-amplitude bending or contact behaviors. Their role is to make the future task space for calibration and evaluation explicit: any later configuration study must remain consistent with the declared segment count, interface positions, collision constraints, and evidence classification used in the present computational comparison. Figure 14 illustrates these candidate arrangements.

#### 5.10.4. Evidence Boundary

This synthesis is a future-validation carrier. No physical prototype, J2 calibration result, nonlinear contact analysis, venue deployment, user study, or cultural-perception validation has been completed. Figure 12, Figure 13 and Figure 14 consolidate mechanisms, engineering variables, frozen negative findings, and unresolved joint sensitivity into a reviewable conceptual architecture; they do not document manufacture, deployment, or interaction outcomes.

### 5.11. Practical Implementation Constraints

Any future physical implementation would require manufacturing and serviceability assessment beyond the present computational model. Multiple segmented interfaces can accumulate manufacturing tolerance through concentricity, alignment, and node-ring dimensional variation; these effects may alter assembly repeatability and the realized interface geometry. No manufacturing-tolerance data or production validation are reported here.

A passively coupled or underactuated interface may accumulate angular play through backlash, friction-surface wear, and cyclic loading. Such play could affect repeatability of interface orientation and service positioning, while stiffness drift could change the response assumed by J1. The present study contains no backlash value, wear test, or calibration-drift measurement. The current Condition C does not specify or numerically model a validated tendon transmission; if a tendon-based transmission is adopted in future hardware, routing, tensioning, wear, and maintenance would require separate characterization.

Additional interfaces may increase inspection, recalibration, and access requirements compared with a simpler rigid linkage. Modular joint cartridges could support replacement and access to internal components, but this is a design intent rather than a validated maintenance benefit. These constraints reinforce the need for J2 torque–rotation calibration, prototype assembly checks, and cyclic service testing before any hardware-level selection or deployment claim.

## 6. Limitations

The biological translation uses literature-based bamboo mechanisms rather than measured local specimens. The work does not establish properties of a specific Xiang–Gan bamboo population. The computational model uses generic PLA assumptions and simplified geometry; it does not reproduce bamboo’s multiscale material architecture.

The kinematic analysis is an idealized geometric model. It omits measured joint friction, backlash, compliance, actuator dynamics, environmental obstacles, and task-level requirements. Its joint ranges are provisional assumptions, and Jtheta is a synthetic computational check rather than physical actuation evidence.

The structural analysis is limited to small-deformation, linear-elastic static and linear-buckling computations for a 500 mm subsystem under the rigid-joint J0 baseline. It omits material and geometric nonlinearity, TPU compliance, passive-joint contact or friction, backlash, tendon/cable compliance, dynamics, fatigue, thermal/moisture effects, human contact, collision safety, and manufacturing variability. The PLA modulus and engineering loads are provisional model assumptions, not characterized properties of a future prototype.

The structural comparison uses J0 rigid-joint constraints as a formal baseline. J1 is an analytical rotational-compliance sensitivity model, using provisional *K*_ro*t*_ values of 0.1–1.0 N·m/°; it is not physical joint validation, and it produced A/C displacement-ranking reversals in four of six specified cases. J2 physical calibration has not been executed. These limitations constrain the generality of H2 but do not alter the numerical validity of the reported J0 comparison under its specified assumptions. No direct extrapolation from the 500 mm subsystem to the approximately 1300 mm concept is made.

The service context is defined by three core Xiang–Gan red-culture venues: the Jinggangshan Revolutionary Museum, the Wenjiashi Autumn Harvest Uprising Memorial Museum, and the Chaling Workers–Peasants–Soldiers Government Site Memorial Museum. Operational requirements—typical visitor flow patterns, spatial constraints of exhibition areas, specific service tasks (guided explanation, object narration, and crowd navigation), and staff–robot interaction scenarios—have been identified from public documentation but have not been empirically verified through on-site observation, staff interviews, or visitor surveys. The cultural significance of Jinggang bamboo as a regional symbol has not been evaluated through structured cultural perception studies with Xiang–Gan participants. All conclusions are bounded to the Xiang–Gan red-culture museum context and are not claimed to generalize to museums in other Chinese regions, to general heritage or cultural sites, or to international museum contexts.

### Evidence Classification and Statistical Limitations

The evidence is classified by source and scope. H1 and H2 are frozen computational hypothesis tests under the recorded comparison models, not externally registered clinical or field trials. The Section 4.2 metrics and LHS analysis are exploratory/post hoc computational outputs. Jtheta is a synthetic computational check. H3, H4-A, H5, and H6 are not tested on independent physical or user data. LHS percentages describe the sampled model space and are not inferential population statistics.

## 7. Conclusions

This study presents a bamboo-inspired morphological design approach for service-robot trunk structures, developed and evaluated within the context of revolutionary heritage museums in the Xiang–Gan region. The contributions and findings are as follows.

This study specifies a reproducible translation pathway from bamboo mechanisms to parameterized engineering variables. It separates biological interpretation, engineering abstraction, computational parameterization, and comparative evaluation. The pathway is proposed for review and future testing; the A/C comparison includes coupled design differences and does not isolate a single causal biomimetic feature.

Under the frozen computational conditions, H1 (composite kinematic coverage) was not supported: C produced 74.1% of A’s model-derived composite coverage (*Ω*_C_ = 885.9 vs. *Ω*_A_ = 1196.0 cm·°). H2 was not supported under the rigid-joint (J0), linear-elastic 500 mm subsystem comparison: C reduced model mass by 7.8% but decreased calculated bending stiffness by 10.5% and mass-specific stiffness by 3.0%. These are conditional model-specific outputs, not physical performance measurements or complete-assembly rankings.

Exploratory analysis found that C retained 95.7% of A’s feasible composite kinematic coverage, while its absolute composite coverage was 25.9% smaller (*R*_re*t*en*t*ion_ = 0.957). The model-derived, active-control-variable-normalized coverage ratio was *R*_DOF_ = 1.436; it exceeded 1.0 in all samples of the expanded post hoc LHS analysis. These findings are hypothesis-generating only, definition-dependent, and require independent physical and computational confirmation. The sample-size and repeated-seed check concerns numerical sampling stability only and does not change the unsupported H1 conclusion.

Joint rotational stiffness was influential in the model’s displacement sensitivity analysis. Under provisional J1 assumptions, four of six primary load cases showed an A/C structural ranking reversal. The J1 result is conditional on an analytical spring model rather than measured connectors; it does not invalidate the J0 calculation, but it prevents any joint-independent hardware ranking claim. No design selection is warranted before J2 calibration and further validation.

All findings are bounded by computational evidence, provisional material and load assumptions, and a 500 mm structural subsystem. The manuscript does not claim a validated physical robot, embodied intelligence, passive safety, cultural-identity effect, venue deployment, or superiority of bamboo-inspired design.

The next unresolved evidence gap is J2 physical calibration of connector stiffness. Nonlinear/contact analysis, prototyping, control testing, venue-specific task study, and cultural-perception research are future work; none has been reported as completed here. Within this bounded context, this study offers an initial computational design route for considering service adaptation together with Xiang–Gan red-culture expression; whether that route communicates cultural value or is meaningful to museum visitors remains to be established empirically.

## Figures and Tables

**Table 1:** Structural comparison of Conditions A and C under S1 (equal envelope and payload) and S2 (equal mass). Values are frozen computational results from linear structural analysis; units and C/A ratios are shown explicitly.

Metric	Regime	Unit	Condition A	Condition C	C/A Ratio
Mass	S1	kg	0.7964	0.7344	0.922
Bending stiffness (*K*_bend_)	S1	N/mm	96.22	86.08	0.895
Specific stiffness (*K*_bend_/mass)	S1	N/(mm kg)	120.8	117.2	0.970
Interface displacement (LC1)	S1	mm	0.004419	0.005147	1.165
Interface displacement (LC2)	S1	mm	0.02536	0.02733	1.078
Interface displacement (LC4)	S1	mm	0.0325	0.03478	1.070
Buckling eigenvalue (LC7)	S1	dimensionless	4893	4459	0.911
Mass	S2	kg	0.7964	0.7964	1.000
Bending stiffness (*K*_bend_)	S2	N/mm	96.22	92.7	0.963
Interface displacement (LC1)	S2	mm	0.004419	0.004791	1.084
Interface displacement (LC2)	S2	mm	0.02536	0.02599	1.025
Interface displacement (LC4)	S2	mm	0.0325	0.03313	1.019
Buckling eigenvalue (LC7)	S2	dimensionless	4893	4822	0.986

**Table 2:** Summary of hypothesis outcomes, evidence levels, and required validation.

Hypothesis	Status	Key Evidence	Main Limitation	Required Validation
H1	NOT SUPPORTED	*Ω*_C_/*Ω*_A_ = 0.741 under the frozen kinematic protocol.	Nominal computational model; no dynamics or joint limits.	Independent data under a prospectively specified comparison.
H2	NOT SUPPORTED	C lowers mass, but also bending and mass-normalized stiffness under frozen linear FEA.	Linear model with provisional inputs and J0/J1 assumptions.	J2 calibration and nonlinear contact analysis.
H3	NOT TESTED	No physical contact-force data.	J2/contact experiment not executed.	Instrumented contact protocol.
H4-A	NOT TESTED	No validated disturbance–recovery control dataset.	*J_θ_* proxy does not validate morphology–control adaptation.	Controlled disturbance-recovery experiment.
H5	NOT TESTED	No user-perception data.	Cultural symbolism is not mechanical evidence.	Pre-registered user study.
H6	NOT TESTED	Post hoc *R*_re*t*en*t*ion_ = 0.957 and *R*_DOF_ = 1.436 motivate a future hypothesis.	The same dataset generated the exploratory observation.	Formulated prospectively for future independent testing after the post hoc exploratory finding; not tested with independent data.

## Data Availability

The computational data supporting the findings of this study, including result tables, model-configuration files, available processed solver outputs, and source data underlying the figures and tables, are maintained in a private version-controlled project repository. The supporting materials that remain available can be provided by the corresponding author upon reasonable request. Large intermediate solver files are not all retained; regeneration is possible only where the corresponding inputs and workflow records remain available. The surviving Run 1 provenance materials comprise a 63-job summary table, a consolidated run log, and the documented load-direction error diagnosis. The full set of per-job Run 1 inputs and solver outputs, and a standalone directional-test execution record, were not located in the archive reviewed. The directional-test source code is retained. No human-participant, user-study, or museum-deployment data were generated in this study.
